# Manufacturing Technologies of Carbon/Glass Fiber-Reinforced Polymer Composites and Their Properties: A Review

**DOI:** 10.3390/polym13213721

**Published:** 2021-10-28

**Authors:** Dipen Kumar Rajak, Pratiksha H. Wagh, Emanoil Linul

**Affiliations:** 1 Department of Mechanical Engineering, G. H. Raisoni Institute of Business Management, Jalgaon 425002, MH, India; 2 Department of Mechanical Engineering, G. H. Raisoni Institute of Engineering and Technology, Pune 412207, MH, India; pratikshawag@yahoo.com; 3 Department of Mechanics and Strength of Materials, Politehnica University Timisoara, 300 222 Timisoara, Romania

**Keywords:** GFRP and CFRP composites, manufacturing techniques, properties, applications

## Abstract

Over the last few years, there has been a growing interest in the study of lightweight composite materials. Due to their tailorable properties and unique characteristics (high strength, flexibility and stiffness), glass (GFs) and carbon (CFs) fibers are widely used in the production of advanced polymer matrix composites. Glass Fiber-Reinforced Polymer (GFRP) and Carbon Fiber-Reinforced Polymer (CFRP) composites have been developed by different fabrication methods and are extensively used for diverse engineering applications. A considerable amount of research papers have been published on GFRP and CFRP composites, but most of them focused on particular aspects. Therefore, in this review paper, a detailed classification of the existing types of GFs and CFs, highlighting their basic properties, is presented. Further, the oldest to the newest manufacturing techniques of GFRP and CFRP composites have been collected and described in detail. Furthermore, advantages, limitations and future trends of manufacturing methodologies are emphasized. The main properties (mechanical, vibrational, environmental, tribological and thermal) of GFRP and CFRP composites were summarized and documented with results from the literature. Finally, applications and future research directions of FRP composites are addressed. The database presented herein enables a comprehensive understanding of the GFRP and CFRP composites’ behavior and it can serve as a basis for developing models for predicting their behavior.

## 1. Introduction

Throughout human history, from early civilizations to enabling future innovations, composite materials have played an important role. Compared to fully dense materials (e.g., steel, aluminum, etc.), composites offer many advantages, some of which are lightweight, high strength and stiffness, excellent vibration damping property, design flexibility, corrosion and wear resistance. Due to these special features, composite materials have spread in our daily lives, starting from the usual household items to complex fields such as the biomedical, sport, maritime and building industries. Moreover, some special applications such as aircrafts, rocket ships and the like would probably not even be able to leave the ground if composite materials were not used. Today, the composites industry continues to evolve, with much of the growth now centered on renewable energy. Wind turbine blades, in particular, constantly exceed the size limits and require high-performance advanced composite materials [1,2,3].

Recently, our mobile civilization has caused increasing attention about environmental protection and transport safety. Major efforts have been dedicated to reducing fuel loss by implementing lightweight materials in automotive, railway, naval, aerospace, etc. [4,5]. Yue et al. [6] manufactured and characterized ecologically sustainable bioplastic composites. The authors used naturally renewable resources, including waste protein cotton powder, dialdehyde starch and natural sisal fibers. All their green composites showed physical, mechanical and thermal properties comparable or superior to ultramodern biomass-based composites. Moreover, Zheng et al. [7] reported improved tensile properties of reinforcing agents based on waste wool fibers, with this being a renewable resource. Reducing greenhouse gas emissions and fuel consumption has become a major focus in the automobile industry. It is predicted that reducing the weight of the vehicle by 10% could lead to a fuel economy of up to 7%, and this means that every 1 kg dropped from the vehicle mass will lead to a reduction of carbon dioxide emissions of approximately 20 kg per 100 km [8,9,10]. Protection and authoritative conditions have enhanced, as this problem grows more significant socio-economically. Due to this purpose, flat structure composites made of lightweight Fiber-Reinforced Polymer (FRP) composites, e.g., Carbon Fiber-Reinforced Polymer (CFRP) and Glass Fiber-Reinforced Polymer (GFRP), have been frequently utilized as energy-absorbing parts in recent vehicles, as they possess moderate density and more leading specific mechanical properties in relation to traditional metals [11,12,13,14].

Over the years, many types of fibers (glass, carbon, aramid, basalt, paper, wood, or asbestos) have been identified or developed for the production of advanced FRP composites. However, the most common fibers for FRP production are glass fibers (GFs) and carbon fibers (CFs). GFs are wonderful at working under high tensile stress, but are inadequate in terms of existing compression owing to their fragile character. On the other hand, plastic materials cannot withstand high tension, but they can very well-tolerate compression loading. Bringing together the distinguished types of materials, GFRP becomes a composite material that takes over both tensile and compressive loads [15]. Due to these behavioral improvements, the GFRP composites find increasing use in electrical, sound and thermal insulation, structure of boats and ships, aerospace applications [16], automotive areas, sports equipment [17], sheet molding compounds [18,19], etc. On the other hand, CFs are fibers composed of principally carbon atoms and hold usual characteristics such as high stiffness, low weight, high tensile strength, high-temperature tolerance, high chemical resistance as well as low thermal expansion. The flexibleness of CFs corresponds to any form, quicker repair, excellent fatigue behavior, least disorder and noise during preparation or establishment. In contrast to other types of fibers, CFs have recorded a better achievement rate and are light in nature [20,21,22]. The hybrid technology of CF and GF is considered to realize the dual advantages of reducing material price and improving long-term durability. To this end, Li et al. [23] and Kar et al. [24] developed novel hybrid composite rods comprised of a unidirectional CF core and a GF shell. The authors studied the tension–tension fatigue performance of the hybrid composite and found that it has superior behavior compared to other traditional FRP composites. The effect of the hygrothermal environment on mechanical and thermal properties of unidirectional CF/GF hybrid composites was investigated by Tsai et al. [25]. The moisture content, the glass transition temperature and the short-beam shear strength are compared and correlated by the authors. Recently, Xian et al. [26], using pultrusion technology, developed three kinds of carbon/glass FRP hybrid rods. They investigated the effects of fiber hybrid mode and rod diameter on the mechanical properties and water uptake behavior.

Many researchers have investigated the failure modes of thin-walled metallic structures, with an appropriate focus on different loading situations. Presently, aluminum alloys are used as a relatively more innovative form of the lightweight metallic materials, and are frequently used to substitute conventional steel in advanced new-developed vehicles [27,28,29,30]. So far, many of the FRP composites have been researched to gradually replace the limited conventional solid materials. Due to the complex collapse mechanisms, including fiber fracture, matrix fracture, film delamination, etc., the failure response of FRP composites is considered laborious and quite difficult to understand [31]. Thus, for safety conditions in different industries, there is an essential requirement to know the main properties and crushing response of FRP composites. However, much of the literature up to now has been research papers, and there are a limited number of review papers. Therefore, the aim of this review paper is to provide a comprehensive literature review of the oldest-to-newest manufacturing methodologies of GFRP and CFRP composites, as well as to present their properties, advantages, limitations, applications and future research directions.

## 2. Glass Fibers and Carbon Fibers

In general, the fibers are obtained by pressing the raw material (in different states) through tight holes, followed by their solidification (under different conditions) and pulling (allows the arrangement of molecules along the fiber axis). There are many types of natural (cellulosic/plant, animal and mineral fibers) and man-made (organic and inorganic fibers) fibers available in the industry. However, glass (GFs) and carbon (CFs) fibers, which belong to the category of inorganic synthetic fibers (SFs), are the most used fibers for obtaining high-performance FRP composites. The two types of mentioned fibers (GFs and CFs) will be presented in detail in the following sections.

### 2.1. Glass Fibers

**Figure 1:**
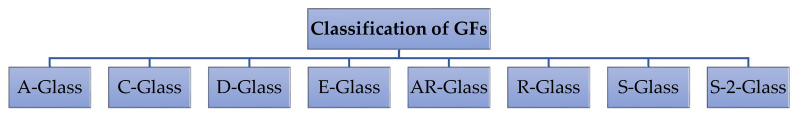
Classification of common glass fibers.

Presently, glass fibers are identified as the most adaptable manufacturing materials among others. They are easily manufactured from crude material, which is accessible in an almost endless supply. Depending on the used raw materials and their proportions to fabricate glass fibers, there are several types of GFs predominantly used in GFRP composites (see Figure 1).

**Figure 2:**
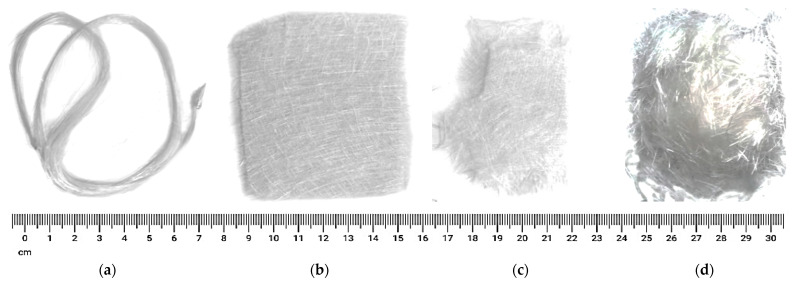
Types of glass fibers: continuously long threaded (**a**), woven and random (**b**), chopped strand mat (**c**) and chopped (**d**) fibers.

For multiple purposes, GFRP composites are widely used in many production technologies. Originally, early Egyptians formed vessels by GFs extracted from heat-modified glass. Continuous GFs were initially fabricated in the 1930s for high-temperature electrical objects. Now, GFs are used in the manufacture of structural composites, in electronics, marine (boat hulls), printed circuit boards, aerospace industry, automobile applications (e.g., rubber tires and lightweight parts) and a wide range of special-purpose products. Low thermal conductivity, high strength, good electrical insulator, elasticity, incombustible, stiffness and protection to chemical injury are the unique characteristics provided by GFs. It may be in the kind of rambling, cut strands, threads, fabrics and mats. Each variety of GF has different properties and is used for different purposes in the formation of GFRP composites. One of the drawbacks of GFs is their moderately low modulus related to carbon fibers. Figure 2 shows the various forms of GF structures, each type highlighting their unique properties [32,33,34].

Young’s Modulus, tensile strength and chemical stability are the GFs characteristics directly mapped upon the fibers. Characteristics such as dissipation factor, dielectric constant, dielectric durability, thermal expansion and volume/surface resistivity are graded on the glass that has been designed into the most specimens. Other properties such as refractive index and density are measured on both bulk samples and fibers. Table 1 shows the chemical composition, special features and main applications, while Table 2 highlights the main properties of various GFs. Special investigations involving animals or humans, and other studies requiring ethical aspects, must list the authority that provided the approval and the appropriate code of ethical approval.

### 2.2. Carbon Fibers

**Figure 3:**
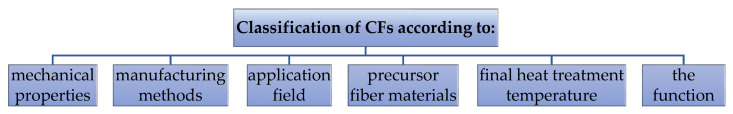
Classification of common carbon fibers according to specific features.

Carbon fibers are fibers of about 5–10 µm in diameter and are more than 90% carbonized. The financial discovery of CFs took place later in the 1960s, when the production of PAN (polyacrylonitrile)-based CF enhanced efficiently, and the carbon yield improved by up to 50%. Nowadays, PAN fiber, viscose rayon, mesophase pitch or petroleum residues (in protected atmosphere) are most widely popular for the manufacture of CFs. The PAN fiber is the source for most manufactured CFs. The main characteristics of CFs (including light weight, high stiffness, high tensile strength, fatigue resistance, good vibration damping, high-temperature stability, high chemical/corrosion endurance, good electromagnetic properties, and electrical conductivity, organic inertness and X-ray permeability, self-lubrication and low coefficient of thermal expansion) have made them very popular in various engineering applications, such as aerospace, automobile and marine transport, military, aerospace antenna and support structure, civil engineering, medical applications (surgery and X-ray equipment, prostheses, tendon/ligament implants) and sporting goods [40]. However, the only drawback of CFs is their high cost when compared with GFs, plastic fibers or naturally available fibers [41]. Figure 3 shows the classification of CFs according to their specific features.

Based on the classification shown in Figure 3, the following CF types can be identified [42,43,44]:

(a) Based on the type of precursor fiber materials: pitch-based CFs, PAN-based CFs, mesophase pitch-based CFs, rayon-based CFs: obtained by pyrolysis to form the first high-strength CF, isotropic pitch-based CFs and gas-phase-grown CFs.
(b) Based on mechanical properties of CFs: general-grade CFs, and high-performance CFs, that include middle, high (>3.0 GPa) and ultra-high (>4.5 GPa) tensile strength type, and low (<100 GPa), intermediate (200–350 GPa), high (350–450 GPa) and ultra-high (>450 GPa) modulus type.
(c) Based on final heat treatment temperature (FHTT): Class-I, high-heat-treatment CFs, where FHTT > 2000 °C, being correlated with high-modulus CFs; Class-II, intermediate-heat-treatment CFs, where FHTT ≥ 1500 °C, being correlated with high-strength CFs; Class-III, low-heat-treatment CFs, where FHTT < 1000 °C, being correlated with low-modulus and low-strength CFs.
(d) Based on different manufacturing methods: carbon fiber (800–1600 °C), oxidative fibers (peroxidation fiber at 200–300 °C), graphite fibers (2000–3000 °C), activated CF and vapor-grown CF.
(e) Based on the function: flame-resistant CFs, load structure using CFs, activated CFs (adsorption activity), CFs used for lubrication, conductive CFs, corrosion-resistant CFs and wear-resistant CFs.
(f) Based on the application field: Commercial-grade CFs: have a large tow, and are associated with a cluster of monofilament thread of more than 24 K (1 K = 1000). To lessen the cost, large-tow fibers of 360 K, 480 K and 540 K were adopted. Aerospace-grade CFs, with a short tow (<12 K), and higher, of 1 K and 3 K carbon fiber tow, recently developed to 6 K and 12 K.

**Figure 4:**
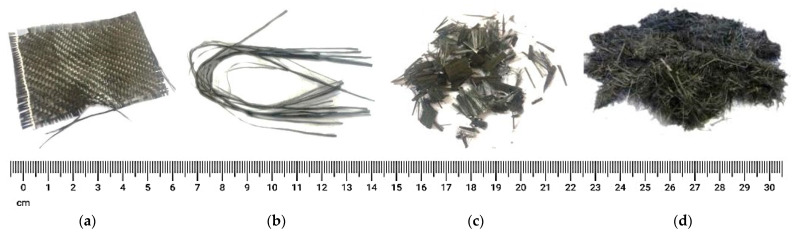
Types of carbon fibers: woven mat (**a**), long threaded (**b**), chopped (**c**) and finely chopped (**d**) fibers.

Figure 4 shows the different types of CFs [32,33,34]. CFs have the most leading tensile strength and elastic modulus (similar to steel) compared to additional conventional varieties of fibers.

Table 3 presents the main physical and mechanical properties of common CFs together with their characteristics and associated applications.

In addition, the CFs are unaffected by alkaline materials or ultraviolet rays. However, their impact properties are less than that of GFs. The CFRPs using ultra-high- and high-modulus CFs are suitable for the aerospace industry because their strength-to-weight ratio is among the highest of all FRP composites. The CFRP composites with high strength and normal modulus fibers are majorly used in the infrastructure industry.

## 3. Manufacturing Methodologies of GFRP and CFRP Composites

Knowing the requirements of a demanding and ever-moving market, the composite structures industry is forced to improve the production methods of FRP materials by developing new types of reinforcements, new resin systems and new combinations of these materials. The main purpose of improving and automating FRP production processes is related to reducing costs and handling time, as well as reducing the weights and sizes of FRP parts.

Throughout the development of FRP composites, in addition to the wide variety of material combinations, different top-level methods are available and feasible for manufacturing the GFRP and CFRP products (e.g., contact molding, matched die molding). The use of these methods ensures sufficient flexibility in optimizing the properties, shape, handling time and production cost of advanced FRP components.

### 3.1. Matched Die Molding

Matched die molding techniques produce highly consistent, net-shape or near-net shape components with low labor cost, and two or more finished surfaces. These techniques include injection, silicone rubber, compression, low pressure, low-temperature compression, transfer compression, resin transfer and structural reaction injection molding.

#### 3.1.1. Injection Molding Process

**Figure 5:**
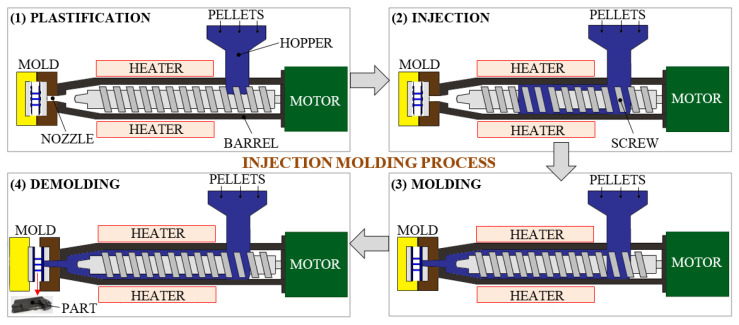
Normal injection molding process cycle: plastification, injection, molding and demolding.

The injection molding (IM) process, also called “thermoplastic injection molding” or simply “thermoplastic molding”, involves injecting a thermoplastic material (molten/softened polymer) under high pressure into a predefined mold. The IM is a reversible molding process. The IM machine consists of several parts, the main ones being: nozzle, mobile plate, heating collar, fixed plate, feed hopper, evacuation system, endless/lamination screw, closing mechanism and console. The mold is composed of two distinct halves: a fixed plate and a moving plate. It is made of aluminum alloys, to the detriment of the commonly used steel, because it allows reducing the investment costs through an easier and faster processing. Once the aluminum mold has been manufactured, the FRP composites in the form of granules/pellets/beads/powder are fed through a connecting hopper and then transported by an endless screw with a controlled heated barrel. The rotation of the plasticizing screw, combined with the proper temperature (of up to 200 °C), softens the used pellets, which are gradually transformed into the molten material. Further, the molten/softened composite plastic material is kneaded and injected under high pressure through a nozzle into the mold by using a hydraulic injection machine. Finally, after cooling, the injected part takes on the desired solid shape and is removed from the mold by means of the ejectors [46]. At this point, a new cycle can be performed. The IM basic process cycle is shown schematically in Figure 5.

This technology allows the duplication of many identical FRP parts of high quality at very low cycle times. The produced components are mechanically equivalent to large and very large series production, ranging from very small parts weighing a few grams (e.g., electronic components) to very large ones of several kilograms (e.g., car body parts). Therefore, the applications of the IM process are very diversified in the lightweight plastics industries, such as aerospace, packaging, automotive, electronic, medical, defense/military, sport and construction [47]. Moreover, the uniformity of the fiber dispersion in the material matrix and the improvement of the fiber–matrix compatibility are obtained by applying surface treatments to manufactured composites [48]. The speed of FRP production and related unit costs are more attractive than other rapid prototyping technologies. This means IM is a very cost-effective and highly efficient form of plastic manufacturing. Furthermore, prior to injection, the IM process makes it possible to add recycled or colored plastic to the virgin material. Of course, certain properties deteriorate, so the recycling process does not ensure the same quality of the FRP parts. Additionally, an advantage not to be neglected is that the obtained parts require an insignificant further post-production machining, while sometimes it is not necessary. However, the obstacle to the IM technology is often the considerable investment in creating the mold. For this reason, the IM process is more cost-effective for large-scale production, but is not recommended for small series of parts (not less than 100 parts). For small series, the SRM process is recommended, for example. In addition, the lifetime of the aluminum mold is limited compared to the steel mold, which is more expensive, and does not allow the production of more than a few thousand parts. Therefore, uniform dispersion of fibers in the matrix, optimal design and increasing the lifetime of the mold, developments of modern methods of recycling injected materials, increasing product quality by finding the optimal pressing force and injection time, respectively identifying the optimal cooling time, are just some of the future research directions that should be developed to increase the performance of injected products.

#### 3.1.2. Silicone Rubber Mold Process

**Figure 6:**
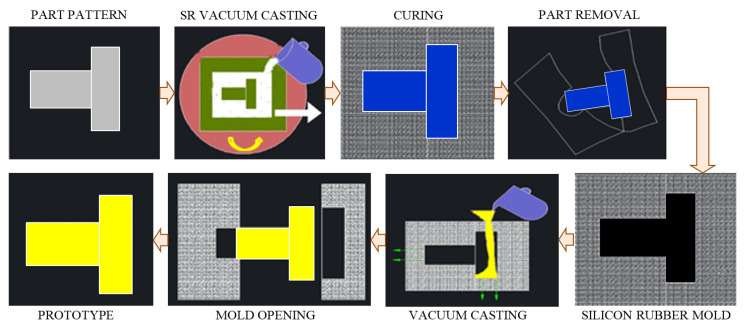
Silicon rubber mold process sequences: part pattern, silicon rubber vacuum casting, curing, part removal, silicon rubber mold, vacuum casting, mold opening and prototype.

The silicone rubber mold (SRM) technology is widely adopted as a casting model material in various top industries, of which the most important are automobiles and electronics. Figure 6 shows the process layouts for fabricating a silicon rubber mold together with SRM process sequences. Initially, the SRM requires the manufacture of an original model by 3D printing or 3D machining. Once the original part is obtained, a silicone rubber model should be manufactured from it. Finally, the outcome, which has the same shape as the original design, can be manufactured [49,50]. For this purpose, Sakata et al. [51] invented the CFRP iso-grid cylindrical shells with the three-axis wire winding equipment practicing the parallelogram pattern of the SRM female model. The axial compressive experiments were proven, and conducted on the reinforcement impact of the layers on the CFRP cylindrical shells. The SRM process allows the high-quality duplication of the model part; however, it has a limited service life, and can only be reused a few times.

Indeed, the mechanical, thermal, physical and aesthetic properties of the created parts are very similar to those injected in series. This is possible due to the large number of existing resins that offer a wide variety of possibilities. Aramide et al. [52] manufactured a FRP composite structure by using GFs and SRM. To serve as a mold release agent, the authors polished the SRM and glazed its surface with hard wax. Finally, to ensure a thin-film distribution of pure resin, a gel coat of unsaturated polyester resin was precisely grazed over the surface of the mold. After gel coat stiffening, successive GF layers and resin are employed. The GF is saturated with resin by rollers and fully wetted. Later, an ultimate sealing film of resin is employed. When the laminate was completely dried, it was removed from the mold and cropped to size utilizing a hand file. On the other hand, Zhao [53] adopted the SRM process for manufacturing CFRP composite springs with low cost, speedy processing time and small material loss. Cushions made of CFRP springs present achievable development in more reliable shock absorption, moderate weight, trustworthy fire safety and more sustained life, without performance degradation.

Moreover, the SRM components can withstand extreme temperatures of heat (+180 °C) and cold (−60 °C), making them an ideal choice for parts under the car’s hood and in the immediate vicinity of engines. Components obtained by SRM technology show high tensile and tear strength, good elongation, excellent flexibility, are fireproof and will not melt. However, surface delamination, shrinkage of the cured component, porosity due to uneven curing, mold flash, flow marks, warping, short shot and burn marks is the list of challenges to be studied in future research to develop an efficient SRM process. All these limitations can reduce the growth of the SRM market size. Furthermore, the large carbon footprint of large-scale silicone rubber production, limitations on the reuse and recycling of such elastomers and single-use challenges will discourage the use of SRM in the near future.

#### 3.1.3. Compression Molding Process

**Figure 7:**
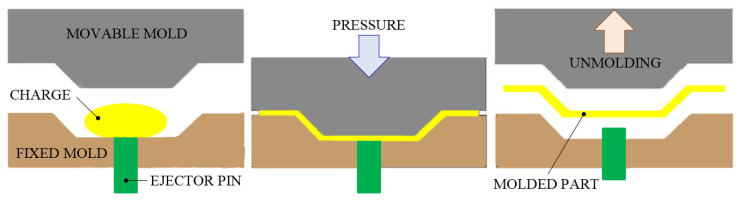
Compression molding process: mold feeding, pressure application, unmolding.

Compression molding (CM) is a FRP manufacturing method in which the preheated (or un-preheated) reinforcement package is initially placed in an open and heated mold cavity. The mold is mounted in a mechanical or hydraulic molding press. The two heated metal mold halves are closed, and high-pressure is applied. The pressure is applied to force the FRP material into contact with mold areas, in order to obtain a desired shape. The external applied pressure and the heat of the mold are maintained until the reinforced material has cured. The molding time, because it depends on the thickness and size of the part, ranges significantly from about a few tens of seconds to the order of minutes. Moreover, the CM process uses different thermosetting resins in a partly cured stage, either in the form of putty-like masses, granules or preforms. Figure 7 presents the CM process, in which charge (FRP package) is pressed between the two heated metal mold halves.

A new class of polytetrafluoroethylene (PTFE)/glass fiber (GF) composite incorporating recycled PTFE/GF, which is initially passed through the CM process, was developed by Xi et al. [54]. The authors observed that the addition of a small amount of GFs (<15 vol.%) will enhance the overall thermal expansion of the new composite, in the Y direction. Moreover, Hameed et al. [55] dressed the chopped stand mat E-GF-reinforced remodeled epoxy composite using the CM technique with various fiber contents (10% to 60%). To release the moisture, the fiber mats were cut into size and heated controlled in an oven at 150 °C. The harder mat was incorporated in epoxy resin. Pre-weighted resin and fiber mats were employed to obtain a composite with 3 mm thickness. The GFRP laminates were compressed in the mold and cured for 3 h at 180 °C. The preserved FRP composite was post-cured for 2 h at 200 °C, followed by a slight cooling to 25 °C for secured final composites. On the other hand, Chauhan et al. [56] show that the invention of S-glass fiber-reinforced vinyl-ester resin composites by varying co-monomers such as styrene, butyl acrylate and methyl acrylate is achievable with the help of the CM process. The composites used in this process are found to have excellent tensile and flexural properties, simultaneous with low densities. 

CM is the simplest form of the SRM process and was first developed to produce FRP components for metal replacement applications, especially from the automotive industry (spoilers, fenders, hoods, scoops). This method of molding offers a short cycle time, easy automation, a high degree of productivity and dimensional stability. Compression-molded FRP parts are characterized by two outstanding finished surfaces and excellent part-to-part repeatability. Actually, high-pressure CM technology is typically used to manufacture high-strength FRP components and high-volume complex parts (flat or moderately curved) in a wide variety of sizes. Moreover, compared with other FRP manufacturing methods (e.g., IM, RTM, TCM, VARTM), it is one of the lowest-cost molding technologies. Furthermore, it relatively reduces wasted material, giving it a major advantage when working with high-cost compounds. In addition, the further processing and finishing costs are minimal. Even so, in order to further increase the quality of the products obtained through the CM process, there are several important challenges that should be taken into account in future research, namely: finding out the exact amount of material needed to make the product, determining the minimum amount of energy and the minimum time required to heat the material, identifying the appropriate heating method, predicting an optimal pressing force, and finally, design of a mold to allow rapid cooling at the end of the process.

#### 3.1.4. Resin Transfer Molding Process

Resin transfer molding (RTM), also called liquid molding, is a well-established FRP composite fabrication process. It is a closed-mold technique suitable for manufacturing high-performance advanced composite parts in medium volumes. Due to its relatively short cycle time, low equipment costs and labor requirements, this method is used to manufacture automotive (truck panels), marine (boat hulls), aerospace and wind turbine blade products. RTM is a low-temperature and low-pressure (usually 3.5–7 bar) process, where a low-viscosity liquid thermoset resin is transferred over previously placed reinforcement materials. The RTM technique is applicable to various types and forms (woven structures, mats, fiber tows, etc.) of fibers. However, it has been reported that the use of GFs and CFs in the RTM process exhibits a positive contribution to the strength and stiffness of FRP composites. 

**Figure 8:**
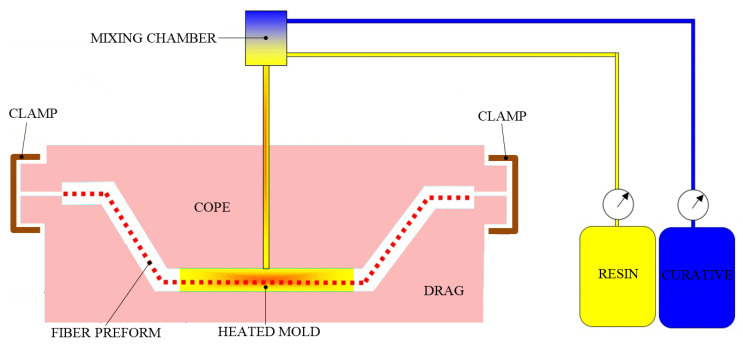
An illustration of the resin transfer molding process for production of FRP composites.

Figure 8 shows the schematic view of the RTM process. The RTM process consists of five main steps: manufacturing the preform, placing the preform inside the mold, filling the mold by resin injection, the curing process and demolding of the obtained part. Initially, for easy removal of the manufactured FRP composite, a release gel is conventionally applied to the surface of the mold. Then, oven-dry reinforcement fibers (typically a preform or a pattern cut roll stock material) are placed in the mold-coated cavity, and the two heated mold plates are closed tightly and clamped to avoid resin leakage. 

Further, resin and catalyst are pre-mixed in specific dispensing equipment, followed by low- to moderate-pressure pumping of the mixture through single or multiple injection ports (depending on the complexity of the part shape) in the fibrous preform. Once the matrix-reinforcement mixture is cooled, different composite tools are used to remove the FRP part from the vented mold. The post-curing process is also necessary to ensure that the resin is completely cured.

The process is highly adaptable and can fabricate components with embedded objects, offering good surface finish (high quality and high dimensional accuracy) of FRP composites. Moreover, due to the low-viscosity fluid and the long reinforcement fibers used in processing, the RTM is particularly suitable for manufacturing low-weight and high-strength FRP composite parts with complex 3D shapes and low resultant voids. Furthermore, a major advantage of the RTM process is the use of low value-added materials (low-viscosity resins and dry fibers) that should not be stored in freezers, thus reducing the handling and material costs. In addition to the mentioned advantages, this technique has a minimum percentage of volatile emissions during processing and single use of low injection pressure. Finally, small parts with very fine details can be produced on rigid metal tooling, while larger parts are manufactured on flexible molds. However, to ensure high-quality parts, the resin flow and resin injection have to be carefully controlled so that all the reinforcements are equally wetted-out. Therefore, to obtain a mold shape that allows the resin to flow evenly to all sections of the part, the flow process requires extensive testing and some advanced fluid dynamics simulations. Two of the most common disadvantages are related to the slowness of the part curing time and the possibility of moving the reinforcement fibers during the transfer of the resin. 

The RTM method produces molded composite components with two high-quality finished surfaces. Fast cycle times can be easily obtained in temperature-controlled tools and the RTM technique can vary from a simple to a highly automated process. In addition, to reduce the internal voids of the FRP composite parts and to improve the resin impregnation, vacuum assistance can be considered to increase mixture flow in the mold. The RTM technique is usually perceived as an intermediate method between the quite slow SLU (having lower tooling costs) and the relatively faster CM method (with higher tooling costs). However, to make the process more reliable and reduce the scrap rate, the RTM process could introduce passive and active control that can take into account the part-to-part variability. This would increase the credibility of lightweight FRP composites and would make RTM competitive for high-volume production. Another challenge in the RTM process is related to the in situ modeling of polymerization. It is difficult to find a modeling system that combines fiber infiltration, resin flow and anionic ring opening polymerization reaction. Therefore, the field of creating a modeling system that requires minimal time and resources is still open.

#### 3.1.5. Vacuum-Assisted Resin Transfer Molding Process

Vacuum-assisted resin transfer molding (VARTM) is a closed-mold composite manufacturing process that has been developed to use effectively during the past two decades. There are different names to describe this process, namely vacuum-assisted resin infusion (VARI), vacuum-assisted resin infusion molding (VARIM), vacuum bag resin transfer molding (VBRTM) and vacuum injected molding (VIM). Moreover, many other developments have been made in the VARTM method, such as vacuum-assisted process (VAP), Seeman composites resin infusion molding process (SCRIMP) and controlled atmospheric pressure resin infusion (CAPRI).

**Figure 9:**
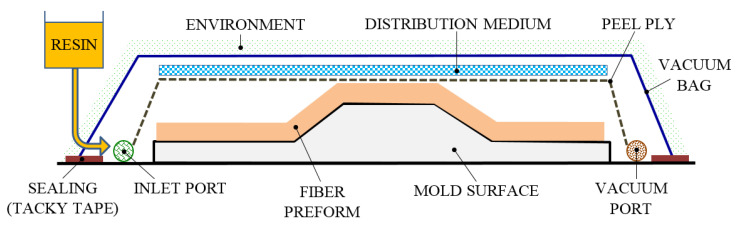
Line diagram for vacuum-assisted resin transfer molding process setup.

VARTM is a variation of RTM, its distinctive feature being the replacement of the upper part of a mold tool with a vacuum bag (VB), and it applies a vacuum to assist the continuous flow of low-pressure infused resin from one side of the mold to the other. The VARTM process involves primarily putting the cloth fabrics or fibers in a preform in the desired configuration. In most cases, these fabrics are held together by a binder and pre-pressed to the shape of the mold. A top (second) matching mold tool is fixed over the first and vacuum-sealed, used as a deformable VB. The pressurized resin is then injected into the mold cavity with the aid of a vacuum. After the impregnation occurs, the FRP composite part is allowed to cure at room temperature, with an optional post-curing sometimes performed. Figure 9 shows a typical VARTM process setup.

This closed-mold method can produce high-performance and different types of composites, especially FRP structures, at a low cost. The VARTM process is widely used for the manufacture of superior and large FRP composite parts, such as in transport and infrastructure. The main advantages of the method are offered by the flexibility for tooling design and material selection, simplicity of changing the geometry of the mold, obtaining complex composites with enhanced quality, god-bearing and structural strength. The VARTM technique can provide up to a 70% fiber to resin ratio with almost zero void content. The main benefit of VARTM is that it lies in the low injection pressures (approximately 1 atm). 

The process parameters in the VARTM technique are volume fraction, part thickness variation and pressure gradient for the manufacture of any type of shape. During the manufacture of large FRP composites, reducing the variation in part thickness over the distance is one of the most challenging tasks in the VARTM process [57,58]. The VARTM process parameters have been modified under various external conditions, such as wet and dry loading, compaction pressure variation, point and line infusions and different resin inlet positions. Yuexin et al. [59] took into account the different orientation of the lay-up and found the thickness and compaction pressure. Song [60] developed an experimental configuration of the VARTM method to identify pressure variation during infusion. Gajjar et al. [61,62] manufactured flat-plate FRP composites using the VARTM process. The authors observed a compaction pressure distribution as the number of layers increased. Moreover, they identified a variation in the thickness of the part along the length with the change of the compaction pressure. Similarly, Vila et al. [63] performed the experiment in wet and dry loading conditions and observed the variation of the volume fraction in relation to the compaction pressure for a different number of layers. Gajjar et al. [64,65] manufactured an antenna reflector using the VARTM method. Yalcinkaya et al. [66] developed a numerical model for determining the infusion time along the length of the part, and compared the compaction pressure distribution with VARTM and RTM methods. Hammami and Gebart [67,68] used wet and dry compaction loading conditions, taking into account the different infusion speed, lay-up thickness and lay-up orientation as process parameters. Song [69] developed an experimental configuration of the VARTM technique to identify pressure variation across the mold. The author considered different resin inlet positions and concluded that the resin inlet position plays a vital role in the pressure gradient of the entire mold. 

Due to the advantages and improved understanding of process physics, the VARTM process is predominantly used in the aerospace, automotive, wind energy, marine and medical industries to obtain products such as aircraft fuselage sections, wind turbine blades, aircraft landing gear doors, large composite panels, low void content parts and high fiber content parts. However, in addition to the advantages of the VARTM process, there are also disadvantages/defects, mainly materialized by fiber misalignment and voids. Many factors, such as the variation of the resin flow pressure or the temperature changes, initiate the formation of voids in this process. All these defects lead to a decrease in strength and modulus, which finally initiates a premature failure of the composite. Konstantopoulos et al. [70] provide a good overview of the solutions and challenges in the field of liquid composite molding procedures, while van Oosterom et al. [71] compare different infusion techniques in terms of process and mechanical parameters. Therefore, to address some of the limitations of the VARTM process, future research directions should focus on several issues, including optimal design and manufacture of the preform (to achieve good control of permeability and compaction), development of a new resin system with viscosity and cure kinetics, improving the filling/infusion time of the resin compartments, development of reusable packaging systems, improving flow and curing control and increasing reliability in detecting and remedying leaks during the process [72].

### 3.2. Matched Die Molding

Contact molding methods offer a lower cost of tools when a single finished surface is required. In addition, these methods allow a shorter product development cycle due to the simplified tool manufacturing process.

#### 3.2.1. Dry Hand Lay-Up Process

The hand lay-up (HLU) process is the simplest and oldest of the FRP composite manufacturing processes. The method is widely used in the low-volume production of large structures from marine (boat hulls and their associated parts), automotive (car body panels), energy (wind turbine blades), transport (large containers) and household (swimming pools, bathtubs, garden pond moldings, architectural work) industries.

**Figure 10:**
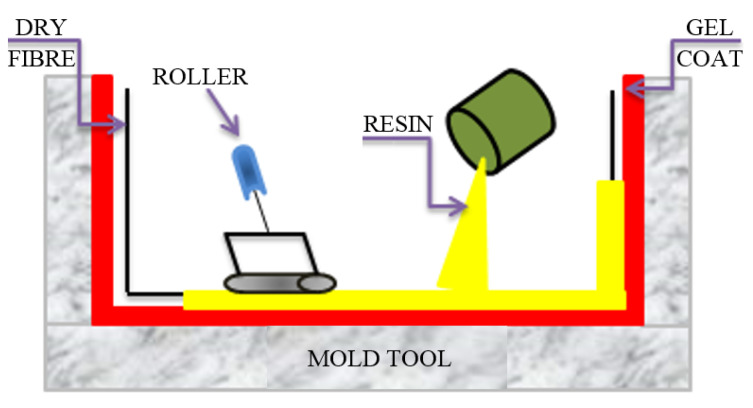
Illustration of the hand lay-up manufacturing process sequences.

The HLU process is shown in Figure 10. Initially, to obtain a high-quality part surface and to protect the mold from moisture, a thin layer of a pigmented gel coat is sprayed on the mold. The applied gel has an anti-adhesive role, facilitating the easy extraction of the obtained composite part. After the gel layer has cured, a desired reinforcing mat is placed on the coated mold. Subsequently, the thermosetting liquid resin (commonly epoxy or catalyzed polyester) is poured onto the reinforcement material (continuous and chopped roving, woven, mat and cloth forms). Further, a manual rolling takes place to remove the air trapped between the reinforcements, ensuring an enhanced interaction between the matrix and the reinforcement. Moreover, the manual rolling also has the role of densifying the FRP composite and thoroughly wetting the reinforcements with resin. Of course, to obtain the desired thickness of the FRP composite, successive additional layers of resin and reinforcement are added. Finally, to avoid the use of an external heating system, a catalyst or accelerator can be used to harden the composite.

Following the HLU process, Suresha and Chandramohan [73] manufactured twisted fabric GFR vinyl-ester composites. The authors used fibers with a diameter between 8 and 12 µm. The resin was incorporated into cobalt naphthenate as an accelerator, methyl ethyl ketone peroxide (MEKP) as a reactant and N-dimethyl aniline as the promoter. Cobalt naphthenate, MEKP and vinyl-ester resin were mixed at the weight ratio of 0.015:0.015:1. A pair of separate fillers was incorporated into resin, such as a 25 mm size of silicon carbide (SiC) and 50 mm of graphite. The woven mats were stacked one above the other and the compound of resin was spread over the fabrics by DHL-U molding. The whole die assembly was pressed at 0.5 MPa constant pressure, by using a hot press. The dimensions of the processed slabs were 3 mm × 250 mm × 250 mm.

The HLU method requires the use of low-cost equipment, being used mainly for FRP components with a high surface area to thickness ratio. Moreover, the simple part processing, high possible degree of shape complexity and easy modification of the design lead to a wide range of part sizes. The HLU technique is ideal for prototypes, respectively for short runs and one larger FRP part or assembly. However, following the open-mold HLU process, the parts have a single finished surface, which requires secondary trim processing. Therefore, the surface detail and surface roughness may be good on the mold-side, but poor on the open-side. Care must be taken that shrinkage increases significantly with a higher resin volume fraction, while gas evolution and air entrapment can develop a weak polymeric matrix and low-strength components. In addition, the amount of catalyst and resin must be accurately measured and appropriately mixed for correct curing times. All of these aspects are a challenge to discover new trends in the HLU process. In addition, as HLU is a high-grade manual handling process, special care must be taken because the flammability and toxicity of the resin is a very important safety issue.

#### 3.2.2. Spray Lay-Up Process

**Figure 11:**
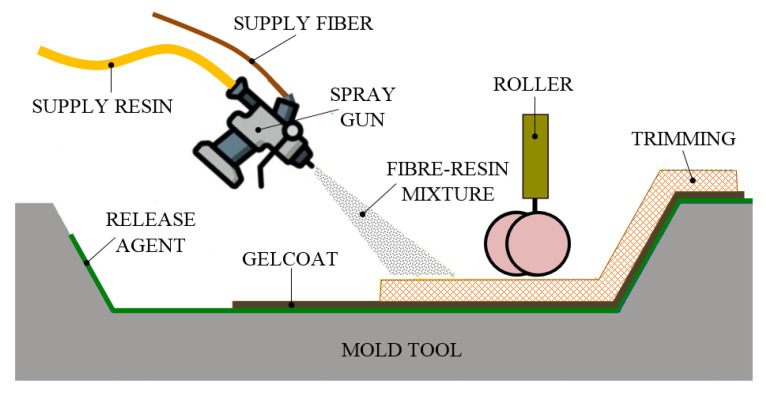
Schematic diagram of the spray lay-up manufacturing method.

Similar to the HLU process in simplicity, the spray lay-up (SLU) technique offers faster FRP production and greater shape complexity. It also uses a low-cost open mold with one finished part surface and curing resin usually at room temperature. Of course, the curing process can be easily accelerated by applying moderate heat. The SLU process is suitable for the production of large FRP composite components, such as bathroom units (shower and bathtubs parts) and ventilation hoods in small to moderate quantities. The method is suitable for low and moderate production [74,75]. The SLU process is illustrated in Figure 11.

The used reinforcements can be continuously long threaded, woven and random, chopped strand mat, chopped or finely chopped. In the case of chopped fibers, their deposition on the mold is performed together with the catalyzed resin by means of a chopper/spray gun. As for the long fibers or woven mat, they are initially placed on the mold and then sprayed. Regardless of the type of fibers used, a manual rolling follows the spraying process, or occurs simultaneously. As in the case of the HLU process, its purpose is to wet the fiber reinforcement and to remove the entrapped air. In addition, to increase the thickness and strength in certain specific areas of the FRP composites, additional woven roving can be often added into the matrix material. Furthermore, pigmented gel coats can also be used in order to produce a colored or smooth surface.

Future research should focus on improving the precision and accuracy of the SLU process from a physical point of view. In this regard, an efficient automation of the process would represent a significant increase both in the quality of the FRP products and in their quantity. Process automation would be ideal, especially for large components, where very high accuracy is required.

#### 3.2.3. Filament Winding Process

**Figure 12:**
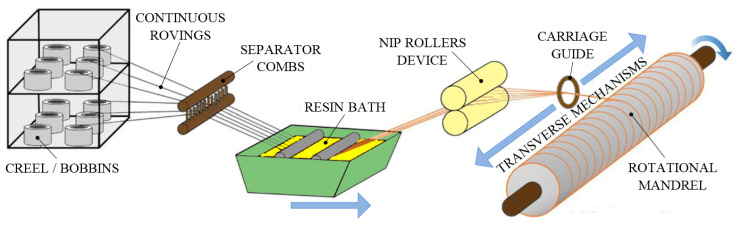
Schematic diagram of a typical filament winding process.

The filament winding (FW) process is a classical manufacturing technique, which is very suitable for automation of the fabrication process. In general, the FW process has three winding patterns: helical, circumferential and polar winding [76]. FW creates open (cylinders, pipes, bicycle forks and rims) or closed end (fuel storage and chemical tanks, stacks, rocket motor cases, pressure vessels, drive shafts) hollow FRP composite structures of incredibly high tensile strength. FW can manufacture axisymmetric as well as non-axisymmetric parts by cross-weaving prepreg sheets, monofilaments and rovings of GF, CF or Kevlar fiber around a rotating mandrel [77]. For non-axisymmetric components (pipe bends), machines with 6 or 7 axes are required, such as those produced by CNC Technics [78]. FW may use either prepreg materials (dry winding) or dry fibers immersed in a resin bath (wet winding) for manufacturing [79]. Other possible applications can be found in aerospace components, military armaments, power and transmission poles, reverse osmosis membrane housings, golf clubs, oars, lamp posts, yacht masts and hundreds of other uses. The basic process of FW is shown in Figure 12.

The first step in the “wet” FW process is to gather the fibers from a set of creels, group them by passing through a textile comb, followed by pulling them through a resin bath. Normally, the resin bath includes a catalyst and liquid resin, but it can also contain additional constituents such as UV absorbers or pigments [80]. At the exit of the resin bath, the rovings of fibers are pulled with a constant tension through a wiping system, such as squeeze rollers, which have the role of controlling the amount of resin deposited on the fibers. Further, the impregnated rovings pass through a ring, comb or straight bar, thus becoming a flat band of fibers. At this stage, the already formed band (or in the case of “dry” FW, prepreg material) is placed on the mandrel, while a carriage system moves back and forth to wind the fibers around the mandrel as the mandrel spins. The carriage and mandrel speeds are adjusted to match the desired winding pattern for the part. After the desired thickness of the laminate has been reached, the mandrel is cured. When the composite resin has fully cured, the mandrel is stripped from the molded part, leaving the hollow composite structure. For larger components, mandrels may be collapsible (segmented or inflatable) for easy extraction from the cured component. On the other hand, for small component volumes, eutectic salts, soluble plasters and low melting alloys are preferred to create the mandrel. FW can be combined with the chopping process and is known as the chop-hoop process.

The factors that control the processing are the temperature of the mandrel, the dipping time and the temperature in the resin, the fibers’ tension and the winding pressure. The wet winding continuous CFRP composites require a mandrel temperature of 70–80 °C, a dipping time of 1–2 s and a dipping temperature of 80–85 °C. For a quality composite, the rovings must be in constant tension during application to ensure a good collimation of the fibers and to reduce the sagging. Thus, a winding pressure of 6–8 MPa and a fiber tension of 8.3–16.6 MPa are recommended, respectively. It is also recommended that for the manufacture of a PEEK-based composite, heated fiber impregnated within the resin and a winding speed of 0.5 m/s must be maintained to obtain the best properties [77]. 

FW is one of the few automated processes for FRP composites’ manufacture and can thus produce high-quality and highly repeatable components at reduced labor content. Since FW is computer-controlled and automated, the labor factor for FW is lower than other open molding composite fabrication processes. A big advantage of FW is that it uses continuous fibers, which leads to good material properties for both stiffness and strength. There is also the ability to orient the direction of the fibers to obtain optimized advanced composites. This process can use almost all types of fibers, from glass (E-Glass, S-Glass, R-Glass) to HS and HM carbon (PAN/Pitch-type CF) fibers. In addition, a wide range of matrices can be used, such as polyester (best cost), epoxy (high-strength, higher temperature capability), vinyl-ester (high-strength, impact and chemical resistance), phenolic (fire-resistance, low smoke) and bismaleimides (higher temperature than epoxies). The main disadvantages are that the process is limited to convex-shaped composites, the mandrel is often enclosed within the winding, fiber cannot be easily laid exactly the length of a composite, mandrel costs for large composites can be high and the external surface of the composite is unmolded, being cosmetically unattractive.

The main controlled variables for winding are resin content, fiber type, wind angle, thickness and tow or bandwidth of the fiber bundle. A very important aspect is given by the angle at which the fiber is wound, because it has a significant effect on the properties of the final FRP part. A lower-angle pattern (helical or polar) will provide longitudinal strength, while a high-angle “hoop” will ensure greater circumferential strength. Voids, micro-cracks, delamination and fiber wrinkles are common defects that appear in the FW parts. In addition, due to the increasing thickness on a rotation mandrel, the FRP composites obtained by the FW process exhibit a lot of residual stresses. It is known that residual stresses can cause either premature failure or dimensional deviations of components. Future research should focus on identifying methods to remove or at least reduce residual stress.

#### 3.2.4. Pultrusion Process

Nowadays, FRP composites are of growing interest in many top industries, and products made using the pultrusion (PT) manufacturing process are not an exemption. As an example, in 2017, the European market (1.1 million tons) for GFRP composites has experienced a steady annual growth of +2% from 2009, of which the PT sector (53,000 tons) showed the fastest growth of +6% [81]. PT is a highly automated process used to manufacture FRP composites with a constant cross-section profile. The “pultrusion” term is a portmanteau expression, combining two different words: “pull” and “extrusion”. The resin-injection pultrusion (RIP) and the resin-bath pultrusion (RBP) processes are the most commonly used PT processes [82]. The PT composite synthesis process is similar to the extrusion technique. The main difference is that the used material is pulled through predefined dies in the PT process, whereas in the extrusion process, the material is pushed through the dies. 

**Figure 13:**
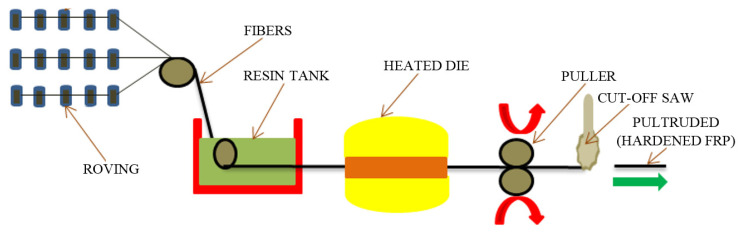
Schematic of the pultrusion process for the fabrication of FRP composites.

PT technology produces FRP composites typically consisting of a thermoplastic or thermoset polymer, reinforced with carbon, glass, aramid fibers or combinations thereof. Most pultruded FRP laminates are developed using rovings aligned down on the main axis of the product. Various continuous fabrics (braided, woven or stitched and knitted), strand mats and bulked or texturized rovings are used to obtain high strength in the transverse direction. Figure 13 shows the PT process, and this involves the following steps: First, (i) the combinations of different reinforcements compose the lay-up of raw material, accurately formed to the required shape. Then, (ii) in order to organize the reinforcements into the profile, the lay-up passes through a predefined guide (pre-die). Once past the guides, (iii) the fibers are impregnated with a resin. In the resin tank, the lay-up is dipped to obtain fully wetted fibers. Further, after leaving the resin tank, (iv) the resin-saturated reinforcements pass through the metal PT die. To control crystallization (thermoplastics solidification process) or polymerization (thermosets curing process) throughout the die, the die is actively cooled and heated. Furthermore, (v) a pulling system guides the profile through the PT die. In order to provide a smooth continuous pull at a stable speed, the puller system has a return stroke that is faster than the pulling stroke. Finally, from the puller system, (vi) the profile reaches the cut-off saw, being cut to the desired/required length. The shape and dimensions of the obtained components follow the shape of the cross of the forming dies, and the shape can be rectangular, circular, square, H shaped, U shaped or I shaped sections.

Kafodya et al. [83] fabricated the FRP sheets with the help of the PT process for underwater applications. Their manufactured sheets presented extraordinary outcomes in the modification of the mechanical properties of CFRP and GFRP composites. Due to the high fiber volume fraction, PT produces structural components with high strength to weight ratio and low labor costs for high volume. The manufacturing process is adaptable to both complex and simple cross-sectional product shapes, eliminating the need for an extensive component post-production assembly. The process speed is influenced by the geometry and profile size. They can vary between 0.02 and 3 m/min for thermoset materials and up to 20 m/min for thermoplastic materials [84]. The fabricated products present high quality, and the productivity is ranked as “excellent” [85]. FRP composite products fabricated under this process are widely used in various industries, such as automotive (structural and complex components of the vehicles with enhanced rigidity, stiffness and lightness), construction (glass fiber reinforcement, carcasses, profiles, stiffening bars), sports and tourism (skis, golf course flagsticks, ski poles and hovel constructions), aerospace (aircraft components), electrical power engineering (dielectric structures, fiberglass and fiberglass profiles) and commercial production (components with enhanced strength).

The quality of manufactured products depends on a lot of factors, of which the most important are the temperature of the die, the preheating method and the fiber passage speed. The advantages of the PT process are represented by good components with very good properties, high production rate and a cost-effective process. Generally, the capital investment for PT is higher than HLU or open-mold processes. The main costs for PT manufacturers are die fabrication and material handling guides’ costs. However, an obvious drawback of the PT process is that obtained parts have limited geometrical shapes, whereas the cross-section may be constant only in the longitudinal direction [84,86]. Moreover, there are very few standards for FRP structures, which makes it difficult to gain approval and market recognition. For example, very recently, the first PT product obtained the European CE marking [87].

The materials used in the pultrusion process are expected to change with the development of new advanced materials. Thus, future research should focus on improving pultrusion machines in terms of both optimizing product quality and handling very large complex shapes. Therefore, the continuous search for innovation and improvement is expected to bring the science of the pultrusion process to a level where newer FRP products can be manufactured with more efficient and cleaner energy options. In addition, increasing the number of standards in the field of pultruded composites’ production could be the subject of future research.

#### 3.2.5. Autoclave Molding Process

**Figure 14:**
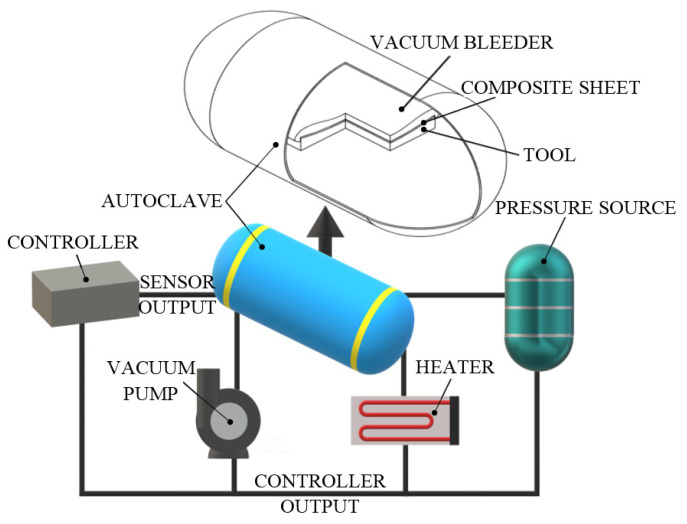
The schematic diagram of the autoclave molding process.

Autoclave molding (AM) is an advanced FRP composite manufacturing technology. AM is similar to the vacuum bag molding (VBM) technique, with the exception that the reinforced material is subjected to higher pressures (5 bar) and denser FRP components are produced. Additionally, due to the use of a closed autoclave, part dimensions are limited compared with the VBM process. Moreover, this technique is somewhat similar to the hot press process, the only notable difference being the way of applying heat and pressure. However, in advanced composites, the autoclave processes are predominantly used, and AM is the preferred process of the aerospace industry. The AM process is relatively expensive, but the FRP products obtained by this method exhibit a versatile fiber orientation, high quality and higher fiber volume fraction. Figure 14 shows the autoclave process which uses coerced steam as the curing agent.

AM is a manufacturing technique that uses a two-sided mold set that forms both outer surfaces of the FRP composite panel. On the upper side of the mold, a flexible silicone/nylon membrane is used, while on the bottom side there is a rigid mold. The continuous reinforcement material (unidirectional tapes and plies, prepreg fabrics or woven cloths), usually pre-impregnated with the resin, can be placed inside the mold automatically (using robots) or manually. In some cases, the lower half-mold is coated with a thin film of resin, and the dry reinforcement is placed on it. Further, the upper half-mold is installed, and vacuum is applied to the mold cavity, where a vacuum pump evacuates the entrapped air. This process eliminates volatile products and air inclusions from the FRP part. The whole assembly is then placed inside an autoclave. Generally, this process is performed at both high temperature and high pressure. The use of inert gas pressure facilitates material curing, a low void content and high fiber volume fraction (densification of the material) for maximum FRP structural efficiency. The vacuum-to-autoclave pressure cycles are chosen to allow maximum air removal without incurring excessive resin flow. The vacuum is commonly applied only in the early stages of the curing cycle, while autoclave hydrostatic pressure (normally in a range of 3–12 MPa) is maintained throughout the entire heating-to-cooling cycles. Following the AM process cycle, the modeled composite is removed sequentially from the autoclave and mold cavity. Autoclave curing allows the manufacture of consistent homogeneous composite products. 

The heat autoclave design considerations are not simple because both radiative and convective heat transfer mechanisms occur. In addition, the thermal resistance of the autoclave is dependent on both the material of the mold and the FRP composite itself. Moreover, the exact time (from tens of minutes to hours) and temperature (up to 180 °C) for curing mostly depend on the type of material that is autoclaved. However, times between 3 and 6 h and temperatures between 120 and 140 °C are the most common ranges for this process. In this regard, Stefaniak et al. [88] used the AM process for manufacturing of CFRP and GFRP composite structures with high dimensional accuracy in the aerospace applications. The authors noted that the autoclave process is clearly superior to other production processes. 

Geometric flexibility in both size and shape is better than for most manufacturing processes. For example, compared to the VBM technique, the AM method produces FRP laminates with more careful thickness control and a lower void content. A disadvantage is that the prepreg content has to be stored in a cold enclosure to prevent resin flow. The capital costs of autoclaves are huge, which forces their use to larger composite structures where these costs are justified. Furthermore, the productivity of the AM process is mainly low because the laying–bagging–demolding cycles consume significant time and labor. All of the above issues (increasing productivity, lowering tooling costs and reducing labor skills) may be considered in detail for future progressive investigations. Even with these deficiencies, in the near future, the autoclave process will benefit from the attention of high-performance, high-quality and low part count applications.

### 3.3. Advantages and Limitations of FRP Manufacturing Methodologies

Regarding the manufacture of advanced CFRP and GFRP composites, the most important aspect is found in the fact that the polymeric material and FRP structure are created at the same time. Accordingly, any defects that are induced during the FRP manufacturing process directly influence the main properties of the FRP composite structure. As previously presented, there are many processes for producing FRP composites (see Section 3); however, all these processes have several features in common. First, the used reinforcements are brought to the desired shape with the help of a tool or mold. Then, in order to cure the resin, the fibers and the resin are brought together using high pressure and temperature. Finally, the desired part is removed from the mold/tool once the resin has cured. However, the selection of the manufacturing technology will naturally have a significant effect on the manufacturing cost, quality and mechanical properties of the part.

**Figure 15:**
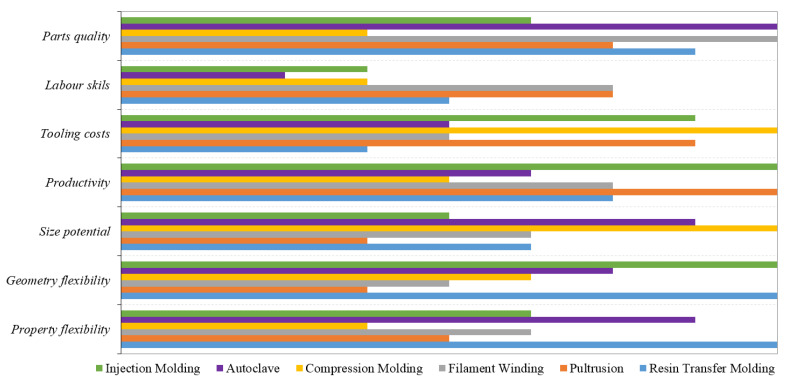
Comparison of different FRP manufacturing processes according to parts’ quality, labor skills, tooling costs, productivity, size potential, geometry and property flexibility [89].

According to Potter [89], an ideal manufacturing technology can be defined as having the following characteristics: minimum material costs (low material storage, low value-added materials and handling cost) and finishing requirements (net shape manufacturing), high productivity (low labor contents, short cycle times), maximum geometrical (size and shape complexity of part) and property (range of reinforcement/matrices types and ability to control main properties) flexibility and reliable and high-quality manufacture (low variability and low reject rates). Nevertheless, there is no manufacturing technology that simultaneously meets all these requirements, which are of course desired by all the top industries. Figure 15 attempts a comparison, based on the aforementioned criteria, of the six most used FRP manufacturing methods (injection molding, resin transfer molding, pultrusion, filament winding, contact molding and autoclave).

It is easy to see that autoclave and filament winding processes offer the best parts’ quality, while compression molding offers the lowest tooling costs and size potential. The best productivity is found in injection molding and pultrusion processes, and the flexibility of properties and geometry is in favor of the resin transfer molding process.

The growing demand for FRP composites leads to a huge consumption of materials and technologies that affect the environment. The current environmental situation has reached a critical point that requires prompt action to decrease greenhouse gas emissions. Energy conversion and storage are crucial aspects of this effort, as they allow the introduction of renewable energy resources. To this end, various groups of researchers have tried to obtain composites from recyclable eco-materials. For example, Zhang et al. [90] manufactured CF composites using biobased dynamic crosslinked matrices from natural epoxidized soybean oil and camphoric acid, without additional chemical modification. The authors observed that the newly developed composites highlight a usable performance at 25 °C. In addition, their laminates can be easily repaired and reprocessed at high temperatures. Moreover, they found that CFs could be completely recycled by degrading composites using ethylene glycol. Their studies show that recycled fibers have maintained almost 100% of the properties of the original samples.

CFs used in the aerospace industry to improve the structural integrity and durability are a major impediment to recycling at the end of the components’ life. Among the major problems are the entanglement of the fibers, short length, as well as their reemployment targets in other FRP structural composites. Therefore, an optimal ecological solution, to these growing problems, would involve the integration of the recycled CFs into a high-value alternative product. Recently, Savignac et al. [91] presented for the first time a new free-standing electrodes formulation that integrates recycled CFs from the aerospace industry. To obtain the desired product, the authors combined the CFs with an active material (LiFePO_4_). LiFePO_4_ material has been chosen for its stability, ultra-fast charge/discharge properties and minimal impact on the environment. In this way, a competitive product (electrodes) was created, and a major environmental problem was solved—collection and disposal of hazardous waste.

## 4. Properties of GFRP and CFRP Composites

### 4.1. Mechanical Properties

The main mechanical properties of fiber-reinforced polymers (FRP), including composites with carbon (CFRP) and glass (GFRP) fibers, should be fully understood prior to designing the composite structures using these types of reinforcements. FRPs using glass fibers are the main reinforcing fiber in all FRP composites. As will be seen in the following, the mechanical properties of FRP structures are subjected to a number of different factors, of which the most important are: fiber and resin type, fiber arrangement (aligned, randomly oriented, braided, etc.) and the percentage of each component. According to [92], the “rule of mixtures” is used to describe the influence of the relative properties of the resin and the fibers. The “rule of mixtures” is given by the following relationship:

(1) $$P_{FRP}=P_{f}V_{f}+P_{m}V_{m}$$

where *P_FRP_* is the studied mechanical property of the new-developed FRP, *P_f_* is the fiber’s mechanical property, *V_f_* is the fiber’s volume fraction, *P_m_* is the matrix mechanical characteristic and *V_m_* is the matrix volume fraction.

Sometimes, when using the “rule of mixtures”, due to the small effect that certain constituents (matrix material or type of fibers) have on the mechanical characteristics of the different FRP composites, they are neglected. For instance, the tensile strength of the FRP is more dependent on the properties of the fibers, while shear strength is more influenced by the resin matrix properties. Comparatively, the Young’s modulus of the FRP composite structure is defined as a role of the moduli of the fiber varieties, while the matrix material influences very little, with this being neglected. In this case, the role of the resin is to transmit the load between the different fibers. Even so, it cannot be said that the varieties of the matrix and its characteristics do not affect the Young’s modulus of the FRP composite at all. For example, Bakis et al. [93] used the “rule of mixtures” to estimate the Young’s modulus and tensile strengths of different hybrid FRP rods. The authors observed that their predictions for the elastic properties were very close to the experimental results, while the strength properties were significantly lower than the experimental ones for most types of FRP rods. This large variation in elastic properties is due to the fact that various organizations fabricate their FRP outcomes utilizing a variety of resin and fibers with different amounts [89]. Tsai et al. [25] performed mechanical property tests and dynamic mechanical analysis on carbon fiber/fiberglass hybrid composites. They found that the main mechanical properties have been retained for the most part (after drying), provided that the moisture absorption does not exceed the saturation point. 

Chen et al. [94] examined the mechanical characteristics of a polyamide66/polyphenylene sulfide (PA66/PPS) compound matrix with several GFs proportions, such as 5%, 10%, 20% and 30% each. The highest tensile strength was observed at 30% *V_f_*, while the flexural strength at 25% *V_f_*. Compared to fiber-incorporated composites, the authors observed the maximum impact strength at 0% *V_f_*. In wear testing, they found that the minimum friction coefficient was around 20% *V_f_* and wear volume was lower, at 30% *V_f_*. The tensile characteristics of plain-weave woven E-Glass/polyester composites, manufactured with various curing pressures (35.8, 70.1, 104 and 138.2 kg/m^2^), were reviewed by Faizal et al. [95]. In order to develop the GFRP composites, they used two different lay-up arrangements, symmetrical and non-symmetrical. Their stress–strain curves revealed that the tensile modulus of GFRP composites was reduced with improving curing pressure for both proportional and non-proportional lay-up. The proportional lay-up was more limited to the stiffness of GFRP composites. Moreover, the ductility enhanced with improving curing pressure for non-proportional, and proportional arrangements declined. Khalili et al. [96] and Soden et al. [97] evaluated the main mechanical properties of different laminates, under various loading conditions. The experimental analyses were conducted using a constant crosshead speed of 5 mm/min and a temperature of 25 °C. Khalili et al. [96] investigated two different materials, one is GRP (Glass-Reinforced Plastic), and the other is FML (Fiber Metal Laminate), while Soden et al. [97] have chosen six types of laminates. Firstly, they observed that the integrity of FML samples with metallic layers is better compared to simple GRP samples. Secondly, due to the different densities, the specific strength of GRP composites is significantly higher than that of the corresponding FMLs. In addition, a comparison of the specific stiffness and stiffness values shows an improvement of the FMLs with respect to the GRP composites [96]. The six composite laminates are considered by the authors to be representative of an extensive variety of FRP laminates confronted in the functional purpose in a variety of engineering applications [97].

Table 4 and Table 5 depict the fundamental properties (tensile and flexural strengths, impact strength, elongation at break) of GFRP and CFRP composites reported in the literature, according to fiber and resin type, curing agent and testing standard. 

Karippal et al. [111] fabricated some epoxy/glass/nano-clay hybrid composites and tested them under tensile and three-point bending fixtures. The main mechanical properties (such as ultimate tensile strength, flexural strength, Young’s modulus, flexural modulus, interlaminar shear strength and Vickers’ microhardness) of the new hybrid composites enhanced with an improvement in nano-clay loading up to 5 wt.% and settled for further loading of nano-clay. Notable improvements in Young’s modulus and ultimate tensile strength achieved in 5 wt.% nano-clay composites were associated with the great combination of nano-clay in epoxy, as reported by SEM images of the sample fractured surfaces. This phenomenon is associated with a better interaction between the matrix material and the nanoparticles. The tension–tension fatigue properties and low-velocity impact behavior of GFRP composites were investigated by Yuanjian et al. [112]. They used two different GF geometries: 0°/90° at 47% fiber weight fractions (Wf) and ±45° at 42% Wf. Their result revealed that the stiffness and leftover tensile strength reduced with increasing impact energy from 0 to 25 J. The outstanding properties were found in up to 10 J tests, while due to improving the testing energy from 10 to 20 J, the preceding properties were highly reduced. The authors observed that the sample deterioration from the impact tests was comparable for the two different geometries. In addition, low impact energy of GFRP composites causes a reduction of the main properties and matrix damage. Their tension–tension fatigue tests were conducted at 1.4, 5 and 10 J impact damage energies. In the stress amplitude (S)–number of cycles to failure (N) curve (S–N fatigue curve or Wöhler curve), the fatigue endurance was slowly reduced and more leading at 1.4 J, with higher stress at ±45° and at 0°/90°. The S–N fatigue curves were abruptly lowered, and they found a more moderate stress value related to 0°/90° geometry. Botelho et al. [113] produced and tested CFR polyamide composites with a varying quantity of layers and thicknesses. The authors obtained a weak interfacial adhesion between the CFs and the applied polyamide with modification in the transverse tensile strength. The polyamide6/6 with more leading carbon fiber content highlighted more eminent shear compression characteristics; therefore, for the thermoplastic matrices’ PA6 composites, the CF volume fraction did not modify the interlaminar shear strength.

**Table 5:** Main mechanical properties of CFRP composites according to different types of reinforcements and matrix.

Reference	Type of CF	Resin	Curing Agent	Testing Standard	Fiber Volume Fraction (%)	Process Type	Sample Thickness (mm)	Tensile Strength (MPa)	Flexural Strength (MPa)	Elongation at Break (%)
[114]	PAN- based carbon fiber	Polyamide6 and polyphenylene sulphide	-	-	-	Injection molding	2	70	-	-
[115]		Polyacrylonitrile	-	GB/T1040–1992	-	Hand lay-up	5	135	-	-
[116]		Polyphenylene sulfide/Polytetrafluoroethylene		Standard–GB 3960-83	-	Mixing and molding	4	113	-	-
[117]		Epoxy	Hardner	-	40	Mixing method	2	3720	-	1.6
[118]		Epoxy	Hardner	ISO 178-1993	-	-	-	-	1154	-
[119]		Epoxy	Hardner	ASTM D-2344	15	Drum winding	0.5		277	-
[120]		Epoxy/phenoxy	Hardner	ISO 180/1A	-	Extrusion	4	-	-	-

In order to achieve a homogeneous mixture of carbon nanofibers (CNF) and SC-15 epoxy resin, Zhou et al. [121] used a high-intensity ultrasonic liquid processor. The optimal carbon nanofibers content was 2.0 wt.%, leading to the highest enhancement in tensile strength. The new-developed CFRP composite produced an enhancement of 22.3% in flexural strength and 11% in tensile strength. The increase of CNF in the matrix also enhanced the fatigue of the FRP composite. Shariatnia et al. [122] introduced a novel processing–manufacturing method to fabricate hybrid (micro/nano) composites. The authors used Cellulose Nanocrystals to assist nanomaterials to integrate Pristine Carbon Nanotubes into CFRP composites without the need to add surfactants or chemical functionalization. It was observed that, compared to neat CFRPs, by incorporating 0.2 wt.% Cellulose Nanocrystals and 0.2 wt.% Pristine Carbon Nanotubes in CFRP composites, the interlaminar shear strength increased by 35%, and the flexural strength by 33%. In addition, the reported results indicate that the incorporation of Cellulose Nanocrystals–Pristine Carbon Nanotubes increases the thermal stability of CFs compared to only Pristine Carbon Nanotubes. They are necessary in FRP composites used in structural engineering applications. 

Castellano et al. [123] investigated the elastic response of anisotropic CFRP composites by ultrasonic immersion experiments, beginning from dimensions of the velocities of ultrasonic waves originating in proper directions. The authors determined, in a non-destructive way, the 5 elastic moduli of an isotropic unidirectional FRP composite material. The physical and chemical changes of a CF composite surface caused by exposure to low-pressure oxygen plasma, as a function of plasma power and duration of exposure, were evaluated by Zhang et al. [124]. The authors noted that O_2_ plasma treatments improved the shear strength of FRP composites from 24 to 27 MPa. There are also changes in both the chemical functionality of the surface and the roughness of the treated composites. Moreover, several researchers evaluated the high/low-velocity impact and static indentation behavior of CFRP and GFRP composites [125,126,127,128]. They observed that the main properties of FRP composites (defined by impact force, damage size and energy absorption) are significantly influenced by the test velocity. 

A challenge in recent years is to develop nanoscale reinforcements that can be used to manufacture CFRP composites for various applications. In this regard, Karakassides et al. [129] used radially aligned graphene nanoflakes, grown directly on CFs, as a novel nano-reinforcement interface. Their results showed that the hybrid CFs not only improve, by 101.5%, the interfacial shear strength between the graphene nanoflakes and the epoxy resin, but also increase the tensile strength of the fibers by 28%. In addition to improved mechanical properties (tensile and shear strength), the authors noticed that both electrochemical capacitance and electrical conductivity improved for yarns, by 157% and 60.5%, respectively. Consequently, all these improvements in mechanical, physical and chemical properties demonstrate the potential of graphene nanoflakes as a reinforcing interface for the cost-effective manufacture of stronger multifunctional CFRP composites.

### 4.2. Vibration Properties

Aluminum alloys are widely used in the aerospace industry for different structural applications [130,131]. Nevertheless, depending on the type of used fibers, the Fiber-Reinforced Polymer (FRP) composites can have a stiffness/weight ratio up to 5 times, while the damping properties exceed 100 times, compared to the aluminum alloys. Therefore, it is not at all surprising that these FRP structures have gradually begun to replace traditional dense metallic materials, especially in those applications where vibrations may occur. For such structures, damping capacity and dynamic modulus are two specific properties that have become attractive for a material in vibration situations. The high damping can mitigate, by dissipating energy, the undesirable effects such as noise and vibrations and their long-term harmful effect on the integrity of the entire structure. On the other hand, the high dynamic modulus provides adequate structural stiffness at a substantially low structural weight, a factor that is of particular consideration in many forms of transport and specifically in the aerospace environment. Due to its energy-dissipating nature, damping significantly provides the impact stability of the material. Furthermore, the structural defects of the advanced FRP composites, such as cracks, voids and delaminations, lead to an abundant increase in damping. On the contrary, in the case of all-metal structures, the damping properties are very low, and they can only be additionally increased by increasing the weight of the product. In hybrid FRP composites, the main vibration characteristics (damping capacity and dynamic modulus) additionally become functions of both the layer stacking sequence and the fiber orientation [132,133,134]. 

Many research investigations have been performed on the vibration characteristics of FRP composite materials. Hemmatnezhad et al. [135] examined the vibration properties of GFRP crystallized composite cylindrical shells, practicing analytical, empirical and statistical investigations. Specially designed filament winding equipment was used to fabricate the continuous GFRP-stiffened specimens. The obtained results of the three varieties of investigations highlight a great deal of outcomes. Some new results are proposed by the authors in courses of natural pulses of vibration and form patterns of new stiffened composite cylindrical shells. Yuvaraja et al. [136] investigated the vibrational characteristics of shape memory alloy (SMA)- and piezoelectric (PZT)-based composites. The authors discovered that the SMA actuator is relatively more active than the PZT actuator since the charge needed for the actuation of SMA is insufficient. In addition, they mentioned that the use of the SMA actuator reduces the huge amplification circuits. A new GFRP composite panel to replace the traditional timber panel was developed by Awad et al. [137]. The authors focused on the available vibration characteristics of the one-step, two-step and eternal GFRP sandwich panels. The 0°/90° orientation provides a higher frequency than the orientation of ±45° in the case of the one-step GFRP panel, while the ±45° orientation provides a tremendous frequency compared to 0°/90° in the case of the two-step traversing panel. Only simple-restraint GFRP panels highlight the most moderate frequency, while the glue-restraint sandwich floor panels present the most leading frequency.

Bledzki et al. [138] investigated the elastic constants of GFRP unidirectional laminates by using the vibration experiment of plates. The authors used two distinct fiber-covering processes. The initial group was covered by epoxy dispersion with amino silane (to improve the fiber/matrix adhesion), while the second group was covered with polyethylene (to restrict fiber/matrix adhesion). They found that the elastic properties were good for the first group of composites and poor for the second. By utilizing several methods (such as Hilbert transform, logarithmic decrement, half-band power and the moving block methods), Naghipour et al. [139] experimentally examined the vibration damping of stuck layered beams strengthened with multiple layers of Glass-Reinforced-Polymer-Reinforced (GRP) glulam composite beams. The half-band power technique enhances the exactness when analyzing the vibration damping of composite materials, maintaining a comparatively enormous level of damping. Besides, the empirical outcomes showed that the increase of GRP reinforcement in the ground surface of the glulam composite beams notably increased their stiffness and strength properties. The vibration properties of CFRP composite pipes, by integrating them with types of active fluids (shear-thickening fluids (STF)), were studied by Gurgen and Sofuoglu [140]. In the vibration tests, a hammer excited the STF/CFRP systems, while the displacements of the CFRP compositions were estimated by an accelerometer to determine the parameters of fundamental dynamics in the modal analysis. The effect has shown that shear thickening fluids’ integration into the composite tubes significantly improves the natural frequency of the CFRP structure as well as providing a higher damping ratio in the STF/CFRP systems. In addition, the damping ratio manifests a good fit with the rheological performance, where the damping property is enhanced as the STF performance becomes stronger in the suspensions. 

Sargianis et al. [141] fabricated carbon fiber (CF) sandwich composites to maintain raised bending stiffness and moderate density, consisting of two weak and hard skin layers and a lightweight core material. The crucial factor in several designs and engineering purposes is lowering the core-specific shear modulus advancement in wave number acknowledgment, which could be accomplished without having to reduce bending stiffness, as witnessed by the authors. Additionally, enhancing the damping properties of a CF sandwich construction, the authors have increased fatigue life, but also degraded the level of noise radiation by diminishing wave number amplitudes. The natural frequency and specific damping range of CFRP and GFRP composite plates, through multiple methods of vibration based on the finite element (FE) method, were investigated by Lin et al. [142]. Their results from the different modes, shapes and fiber orientations revealed a lot of prominent twisting. The observed twisting is more pronounced than for those in which the bulk of the strain energy is saved in tension/compression (in the fiber) and not tension or shear (in the matrix). The obtained outcomes recommended that the FE method employing the damped component model is a great general-purpose instrument for the examination of constructions manufactured from composite materials.

The vibration features such as structure, frequency and amplitude should be investigated at the time of manufacturing or machining of GFRP and CFRP to determine the effect of wear on parts used in a marine or other related application.

### 4.3. Environmental Properties

The integrity and durability of Fiber-Reinforced Polymer (FRP) composites in various environments can be affected by the different and specific properties’ responses of its elements (e.g., polymer matrix, fiber) and by the current interface between the polymer matrix and fiber. All FRP components and structures are exposed to a certain environment during their long-term utilization, but their responses to degeneration depend on the characteristics of the atmosphere. Low or high temperatures, water immersion, humidity, ultraviolet (UV) exposure, alkaline environment, saltwater, etc., can identify the main environmental conditions. More severe operating conditions of advanced structural FRP composites can be found in cyclic exposure (freeze–thaw cycling, high humidity cycling, etc.) or if a combination of different factors occurs [143]. 

The physical and mechanical characteristics of FRP composite structures thus modify the presence of precipitation at the fiber–matrix interface, and likewise can alter the interfacial adhesion. Furthermore, the energy correlated with UV exposure is competent in scattering the chains of molecules in the matrix and can begin material degeneration. Therefore, the fiber–matrix boundary surface is a consequence of the linking of FRP ingredients—it has its chemistry and morphology and highlights the crucial area in FRP composites. Exposure of FRP to low temperatures may cause a ductile-to-brittle transition, which leads to the initiation of micro-cracks. Despite the type of use, once micro-cracks have been created within FRP materials, the sincerity of the composite construction is automatically negotiated. On the other hand, exposure to high operating temperatures can lead to the occurrence of the softening phenomenon and to the degradation of the main properties [144,145,146,147].

The material responses of FRP composite structures subjected to different environmental effects have been well-reported [143,144,148,149,150,151,152,153,154,155,156]. Ray [148] observed that by changing the humidity cycle, the moisture absorption rate changes with a constant temperature environment, and also depends on the sort of matrix resin as well as the weight ratio of ingredients. The influences of varying temperature and humid provisions have a limited influence on diminishing the interlaminar shear strength conditions for both epoxy and polyester systems. Thermal shock impacts are not so remarkable, and this could be related to the appearance of moisture over thermal cycling. The influence of thermal and cryogenic treatment on hygrothermally modified GFRP laminated composites was examined by Mishra et al. [149]. The important property of the fluctuations in the interlaminar shear strength values, which was observed by the author, was that the post-hygrothermal methods increased the rate of desorption of moisture by performing this treatment before the appearance of thermal or cryogenic conditioning. The amount of de-moisturization of the hygrothermal GFRP composites due to thermal exposure is reported to be inversely associated to its interlaminar shear strength, independent of the fiber-weight fractions. Araujo et al. [150] studied the water sorption performances of fiberglass wastes/polyester resin composites, varying distinct percentages of recycled fiber wastes (20%, 30%, 40%, 50% and 60%). The test specimens were submerged in distilled water at distinct time intervals up to 600 h, while a water sorption versus time curve was plotted. It resulted that the water sorption reduced with increments of fiber-waste proportion in the composite, and the least water sorption was seen for the polyester/fiberglass wastes (40%) composite. 

The effects of the exposure of FRP composites to the environment and the long-term retention of properties are significant concerns for various engineering applications, in which the lifespan can last several decades and no or little maintenance is expected. In this regard, Tsai et al. [25] investigated the influence of the hygrothermal environment on the mechanical and thermal properties of CF/GF hybrid composites. They found that the glass transition temperature and the shear properties were sensitive to the effects of the hygrothermal environment, and the values of both properties decreased with increasing absorption. Moreover, after microscopic inspection, it was observed that if the absorption was lower than the saturation, the water-soaked samples did not show cracks. Recently, Xian et al. [26] investigated the effects of rod diameter and fiber hybrid mode on the water uptake behavior. The carbon/glass FRP composite hybrid rods were exposed in the laboratory freezing–thawing cycle (exposure temperature: −25 – +40 °C, exposure medium: distilled water, duration: 12 h) and outdoor environments (exposure temperature: actual outdoor temperature in Harbin, Heilongjiang, China, exposure medium: air, duration: 0, 45, 90 and 360 days). The water uptake tests were conducted to obtain the long-term life evolution, while the thermal property tests were performed to reveal the degradation mechanisms. The authors obtained that the temperature and temperature alternation effects on saturated water uptake time and acceleration factors were remarkable compared to salt concentration and hydraulic pressure. The alternating temperature contributed to an additional degradation rate of up to 31.2% of the stable strength retention compared to the constant temperature. In addition, they found that the rods with a smaller diameter and random hybrid fiber mode had superior corrosive resistance. 

The environmental effects (ultraviolet radiation, hygrothermal exposure, thermal shock aging and salt spray) on the thermal properties of glass fiber (GF) reinforced by poly (ether-imide) (PEI) composites were investigated by Botelho et al. [151]. The experiment was carried out with diverse temperatures at corresponding moisture of 90% for 60 days beneath seawater. The moisture absorption performance of PEI/glass fiber laminates was frequently reliant on temperature and corresponding moisture. The moisture absorption curve advised that the weight addition be originally raised linearly concerning time. The highest moisture absorption of about 0.18% was found after 25 days. Moreover, Ellyin et al. [152] raised the temperature on the mechanical characteristics of glass-fiber epoxy composite tubular specimens, and studied the consequences of precipitation penetration and susceptibility. By varying the temperature range (20 and 50 °C), the GF composite tubes were immersed in distilled water. The experimental tests were carried out for 4 months, and the time versus moisture absorption curve recorded that a 0.23% weight accumulation was detected at 20 °C and 0.29% at 50 °C. Besides, the consequences of water and alkaline environments on the interfacial link concentration among the concrete and the rebar and the strength and stiffness of the GFRP rebars under different temperatures (20–120 °C) were investigated by Abbasi et al. [153]. Various compounds such as GF/isophthalic polyester, GF/vinyl-ester and GF/urethane-modified vinyl-ester were used in this test. Their experimental program was managed at different temperatures (20–120 °C for 30, 120 and 240 days) under normal alkaline and water environments. The GF modulus and composite’s strength were reduced in the alkali atmosphere at tremendous temperatures. 

Zhou and Lucas [154] investigated the effect of a water situation (H_2_O) on moisture immersion features of a unidirectional T300/934 graphite/epoxy composite material by the determination and analysis of hydrothermal-induced expansion, weight difference, surface mass destruction and covering crack development. Samples were submerged in distilled water at different temperatures (45, 60, 75 and 90 °C) for more than 8000 h. Notable dimensional variations occurring from moisture-induced extension were recognized in the width and thickness regions of the graphite/epoxy composites. The unidirectional GF-reinforced and glass-carbon/epoxy hybrid composites were studied by Shan and Liao [155] under tension–tension fatigue tests in the air and in distilled water at room temperature (25 °C). Their specimens exhibit better retention in fatigue lifetime (up to 10^7^ cycles) in water than the corresponding all-glass composite samples. The authors observed that by hybridization with a proper quantity of carbon fibers, protection to environmental exhaustion degradation of GFRP could be significantly improved. Dickson et al. [156] compared the fatigue behavior of carbon fiber/PEEK composites with carbon/epoxy material of similar construction, particularly concerning the effect of hydrothermal conditioning strategies. Sheets of both materials were of 0°/90° lay-up, and they were examined in replicated tension at 0° and 45° to the major fiber axis. The authors observed that due to the naturally superior properties of the thermoplastic matrix, the fatigue response of cross-plied carbon fiber/PEEK in the ±45° orientation is more beneficial than that of carbon/epoxy composites. Combinations of both materials could demonstrate to have considerably more immeasurable fatigue protection than comparable carbon/epoxy composites.

### 4.4. Tribological Properties

The dominance of Fiber-Reinforced Polymer (FRP) in industries has increased the need for scientific research to develop new reinforced composites and evaluate their main properties. FRP composite structures are used on an increasing scale in engineering applications (e.g., grasp pivot box, brakes, cranes, excavators, bearing, medical equipment, etc.), in which tribological properties highlight a significant importance. Among the different analyzed properties (mechanical, physical, thermal, environmental and vibrational), tribological characteristics of the composite structures help to understand their wear and friction behavior. Fiber volume fraction, sliding speed, connected load, surrounding condition, sliding time and filler material activity temperature are the elements that for the most part influence the tribological properties of synthetic FRP composites. The addition of different types of fillers (including GFs and CFs) in the polymeric matrix tends to enhance the tribological behavior of FRP composites through diminishing the coefficient of friction and the corrosion rate.

There are many research papers published by numerous scientists related to the tribological characteristics of FRP composites [157,158,159,160,161,162,163,164,165,166]. Srivastava and Wahne [157] prepared, by the hand lay-up method, GFRP composites filled with mica and tricalcium phosphate (TCP) particles. TR-20LE wear and friction testers were employed to examine the performance of random direction short E-glass fiber-reinforced epoxy resin composites. The outcome has shown that the shreds as the fillers allowed to significantly enhance the main mechanical characteristics, and wear protection of the E-GF as the fillers improved the adhesive bonding strength among the fiber and the epoxy resin. The tribological behavior of the nanoparticle-filled GFRP composites was investigated by Srinivasan et al. [158]. The GFRP composite structures filled with nano-alumina (Al2O3) particles showed better friction with excellent wear performances, and also the fiber break was practically excluded from the wear performance of GFRP filled with a 2% volume fraction nano-Al2O3. Furthermore, the influences of velocity and load on the sliding wear properties of glass fabric-epoxy (G-E) composites with various fillers (oxide and rubber particles) were examined by Kishore et al. [159]. By using a block-on-roller test configuration, the authors considered the sliding velocity between 0.5 and 1.5 m/s at three various loads of 42, 140 and 190 N. The oxide particle-filled composite highlights better wear resistance compared to rubber particles at low load situations. However, rubber shreds had fine wear resistance compared to the corresponding oxide shreds when more leading loading conditions were taken into consideration.

Kishore et al. [160] analyzed the wear performance of the GF-reinforced epoxy composite under dry sliding conditions with the help of a scanning electron microscope. The diverging analysis parameters were utilized, such as load 20–60 N, velocity 2–4 m/s and sliding distance 0.5–6 km, respectively. The pin-on-disc test outcome revealed that raising the load and velocity boosted weight loss. The wreckage rate was lower for shorter distances and higher for longer distances. The wear and friction properties of the chopped strand mat GF 450 g/m^2^ reinforced polyester composite were investigated by Yousif et al. [161]. The tribological properties have been assessed under wet contact conditions upon a polished stainless-steel counter-face, using block-on-ring and pin-on-disc techniques. The authors used two distinct fiber adjustments (parallel and anti-parallel) for specimen preparation. The experimental result highlighted that the appearance of water raised the roughness value in both parallel and anti-parallel adjustments; furthermore, parallel adjustment had less wear and frictional resistance than anti-parallel adjustment. Moreover, Mohan et al. [162] studied the sliding wear performances of Jatropha oil cake filler fusion into GFRP composites for various loads (10 and 20 N). The results of the pin-on-disc setup show that the wear loss enhanced with the addition of sliding distance. By applying a load of 10 N, at a 2000 m sliding distance, the load wear loss was recognized. The Jatropha oil cake-filled GFRP composite had a high coefficient of friction and high-grade wear resistance at different sliding distances.

Chauhan et al. [163] investigated the influence of differences in applied normal load and sliding velocity on the sliding and friction wear performance of a glass-vinyl-ester composite (G-V). The authors estimated the weight shift and analyzed the surface characteristics of worn samples using SEM. They used three distinct combinations of samples, such as GF + vinyl-ester + methyl acrylate, GF + vinyl-ester + styrene and GF + vinyl-ester + butyl acrylate. The experimental tests were performed under the dry sliding condition at different sliding velocities (1, 2, 3 and 4 m/s) and various loads (10, 20, 30 and 40 N). A more moderate coefficient of friction and a more leading specific wear rate at a lower sliding speed was observed for GF + vinyl-ester + butyl acrylate. The tribological performances of nano-TiO2 particle-filled polyetherimide (PEI) composites, reinforced with little CFs and greased within with graphite flakes, were studied by Chang et al. [164]. The distinct wear rate of PEI was lessened to 7.7 × 10^−7^ mm^3^/Nm at 1 MPa and 1 m/s standard testing condition. Besides, nano-TiO2 diminishes the contact temperature and the frictional coefficient of the nanocomposite. Xu et al. [165] fabricated the carbon/silicon carbide composites by the chemical vapor infiltration process in the application of aircraft brakes. The coefficient of friction and friction stability of C/SiC composites was significantly enhanced by improving the carbon proportion and material density. Werner et al. [166] investigated the influence of vapor-grown carbon nanofibers, of average diameter 150 nm, on the wear behavior of semi-crystalline poly (ether ether ketone) (PEEK). It was noticed that with the uniform corrosion provisions during their entire life at a price related to that of conventional PEEK composites, the carbon nanofibers were seen to significantly reduce the wear rate of PEEK.

### 4.5. Thermal Properties

The continually increasing use of fiber-reinforced materials for extensive architectural purposes demands a more immeasurable perception of the main thermal properties (thermal conductivity, specific heat capacity, mass or density, etc.) of FRP composites. The high durability of FRPs ensures that these composites have a more stable thermal behavior in terms of thermal conductivity and do not suffer weathering and aging. It is well-known that thermal conductivity is the characteristic of a material that explains its capability to transfer heat. The thermal conductivity of all polymeric materials is low, which means that FRPs should be good heat insulators. Thermal conductivity of an FRP composite is a function of several factors, of which the most important are fiber and matrix type, fiber volume fraction, fiber characteristics, the regulation of heat flow, matrix–fiber interaction and assistance temperature. The distribution of the temperature fields in the FRP structures can only be determined if the thermal conductivity of the environment is known, while for any engineering material, a low thermal expansion is ideally needed. Identifying the thermal responses in FRPs performs a decisive part in their appearance; therefore, perfect thermal data of FRP composites are required.

The specialized literature is not very rich in terms of FRP thermal properties [167,168,169,170,171,172,173,174]. For estimating the thermal conductivity of some composite materials, different theoretical approaches have been addressed by Caruso et al. [167], Hashin [168], Springer and Tsai [169] and Muralidhar [170]. Gowayed [171] studied the thermal conductivity of a CFRP composite under both transverse and axial directions. With the addition of fiber volume fraction, he obtained a non-linear increase in the thermal conductivity, finally stating that no theoretical model can predict this behavior. Recently, Yung et al. [172] developed the void glass microsphere (HGM)-filled epoxy composites, with filler proportion varying from 0 to 51.3 vol.%, to adjust the dielectric characteristics of the epoxy. As the HGM content increases, the dielectric constant and dielectric failure of the composites decrease, which are crucial for the performance of a superior high-frequency device. Moreover, the authors observed an improvement in the glass transition temperature and the coefficient of thermal expansion. Further, to increase the thermal behavior of a chopped strand, E-GF-reinforced modified epoxy composites with different volumes of fibers (10%, 20%, 30%, 40%, 50% and 60%), Hameed et al. [55] used poly (Styrene-Co-Acrylonitrile) to transform diglycidyl ether of bisphenol-A class epoxy resin restored with diamino diphenyl sulfone. A nitrogen environment was adopted for the testing at the temperature scale from 30 to 900 °C. The thermogravimetric analysis (TGA) revealed that a 60% volume of fibers presents greater thermal stability and an increase in the temperature of degradation (from 357 to 390 °C).

In the absence of filler, Lopez et al. [173] examined the probability of reusing GF waste occurring from the TGA report of E-GF waste polyester composite. The TGA and differential TGA results revealed that the degeneration temperature changed from 209.8 to 448.7 °C, and mass loss changed from 1.8 to 4.4 wt.%. The new-developed CFRP composites, by the high-performance clay/epoxy nano-composite and woven CF fabric, were manufactured and investigated by Phonthammachai et al. [174]. The nitrogen atmosphere is used for the temperature measurement of the samples at a temperature from 25 to 800 °C. To scrutinize the thermal stability of clay/epoxy CFRP, the heating rate range was 2, 5, 10 and 20 °C/min. TGA was performed with a Q500 TGA analyzer instrument and revealed that both neat epoxy and 0.6 vol.% salinized clay/epoxy CFRPs exhibit high thermal stability with a degradation temperature of 370 °C. The degradation temperature was consequently raised with the heating speed, from 350 to 400 °C, at 2 and 20 °C/min. All these favorable thermophysical and thermomechanical properties of FRP composite structures are based on the matrices’ low density and fibers’ high strength. 

## 5. Applications of GFRP and CFRP Composites

The limitations of conventional metallic materials (e.g., steel, aluminum, etc.) have led to a large increase in the use of Fiber-Reinforced Polymer (FRP) composites in various engineering applications. The common applications of FRP composites are continuously diversifying due to their attractive material properties (see Section 2, Section 3 and Section 4). Following these causes (limitations of conventional materials and attractive properties of advanced composites), the applications of the FRP can be grouped as follows [175,176,177,178,179,180,181,182,183,184,185]:

▪ Space: satellites, space centers, launch vehicles, spaceports, remote manipulator arm, payload bay doors, antenna struts and ribs, high-gain antenna, etc.
▪ Aircraft: floorings and panels of airplanes, drive shafts, elevators, rudders, landing gear doors, bearings, etc.
▪ Marine: offshore construction (seawater piping, stairways and walkways, firewater piping, grating, fire and blast walls, cables and ropes, storage vessels, etc.), valves and strainers, fans and blowers, propeller vanes, gear cases, condenser shells, etc.
▪ Automotive: body panels and doors, engine blocks, drive shafts, automotive racing brakes, clutch plates, filament–wound fuel tanks, push rods, bumpers, frames, valve guides, rocker arm covers, etc.
▪ Civil engineering: the execution of new advanced structures (roofs, plate and shell elements, linear elements, pipes and tanks, folded structures, etc.) and the rehabilitation of existing metallic and concrete structures such as buildings, bridges, pipelines, masonry construction, etc.
▪ Sport industry: golf club shafts, tennis rackets, bicycle framework, fishing rods, etc.
▪ Electrical and Electronics: power line insulators, fiber optics tensile members, lighting poles, etc.
▪ Chemical Industries: racked bottles for fire service, composite vessels for substances, mountain climbing, ducts and stacks, underground storage tanks, etc.
▪ Medical applications: tissue engineering (blood vessels, bone, oral tissues, skin, etc.), wound dressing, dental resin-based composites, etc.
▪ Highway structures: sound barrier, bridge deck, beams, stringer, rebar, abutment panel, dowel bar, signboard and signpost, pole and post, drainage system (pipe, culvert), guardrail system, etc.
▪ Agricultural and industrial buildings: for structural and nonstructural elements.
▪ Renewable energy: wind turbine blades.

**Figure 16:**
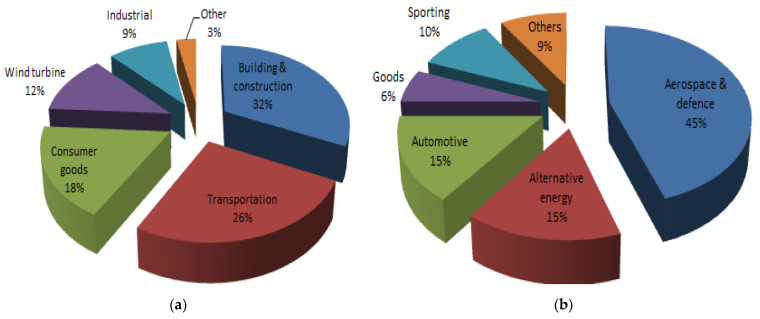
The main applications of glass (**a**) [187] and carbon (**b**) [188] fibers: automotive, alternative energy, aerospace, transportation, building, sport and others.

Figure 16a shows the worldwide application of GFs in various sectors. GF is in high demand due to its excellent tensile strength, low cost and easy availability. Today’s global market has given very much importance to the GF as it is used in various applications, such as the structure and architectural division. Particular areas of the world have generated a demand for private structures due to the increasing communities. In the coming years, GFs will be used for manufacturing furniture and fixtures, tubes, liquid accommodation tanks and wallboards for this sector [186].

CFs are used, as shown in Figure 16b, largely in applications demanding high-stiffness properties exceeding the tensile modulus of glass or aramid fibers. They are also employed in applications where aramid fibers’ poor compression resistance and sensitivity to moisture regain have produced lamination failures.

**Figure 17:**
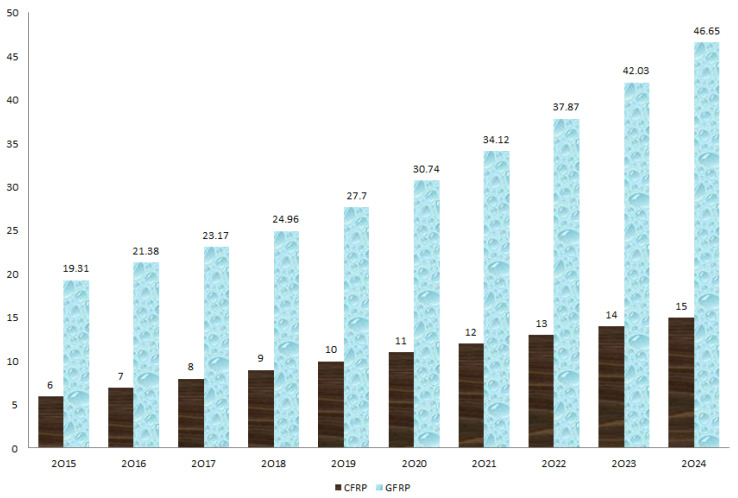
The growth diagram of the CFRP and GFRP composites market from 2015 to 2025 [198,199].

Presently, the price of CFRP composites has dropped, presenting them as more attractive for use in many applications. The CFs are an ideal choice for aerospace and defense applications as they provide excellent strength, durability and resistance, as required. Conventional metal structures are being replaced by CFRP composites in aircraft, due to their lightweight and strong design structure. In the defense industry, CF is also used in missile defense, ground defense and military marine defense. Figure 17 presents the continuous growth of the CFRPs and GFRPs market from 2014 to 2025 in percentage of billion USD.

Chloride compounds are one of the major components of water treatment plants that also causes corrosion of various materials. Therefore, these materials are replaced by CFRP and GFRP composites. Sulphate attacks, abrasions and steel corrosions are major threats to the sustainability of the marine structures. CFRPs and GFRPs have high fatigue endurance, high strengthening properties and prevention from ruptures, and these properties will help CFRPs and GFRPs to be the major players in marine structures and waterfronts [189,190,191]. Many countries, such as the United States, Western Europe, China, Japan, Taiwan, South Korea, South and Central America, Eastern and Central Europe, etc., are using the CFRP and GFRP composites. Significant improvements in technology and processing have developed the need for high-performance CFRPs and GFRPs. The introduction of higher-volume and lower-cost fibers, linked with accretions in productivity, has decreased the manufacturing costs of GFRP and CFRP composites. Considering that cost is an important factor influencing demand, maintained advancements in production, along with enhanced availability, are required to promote rising consumption in all areas and applications [192,193,194,195,196,197].

In the United States, CF composites used will likely develop in mass-produced automobiles, contributing lower weight, extra fuel efficiency and more economical emissions; however, more expensive costs will proceed to limit popular use [200]. Currently, Chinese CF production has expanded and made advancements in its capital presence and quality. Despite this, difficulties continue, such as the requirement for producers to have more comprehensive collaboration with potential customers, the need to establish product standardization and parent supply and quality issues. CFs will grow well in the aircraft and aerospace market divisions, as well as sporting goods [201,202,203]. The United States, Western Europe and Asia import the CFRP and GFRP composites from Japan in a very large amount. Other Asian countries, including South Korea, India and Taiwan, will undergo a continued increase in CFs’ consumption [204].

## 6. Conclusions and Future Trends

The manufacturing methodologies, properties (mechanical, vibrational, environmental, tribological and thermal), advantages, limitations and main applications of GFRP and CFRP composites have been reviewed. The important application of these composites has been highlighted along with their failure modes. Multiple development technologies were utilized for developing the GFRP and CFRP composites, with several climatic requirements. Flexural strength and ultimate tensile strength of the GFRP and CFRP composites were enhanced with an improvement in the CF and GF content of fiber weight portions. The Young’s modulus and elastic strain of the GFRP and CFRP composites increased with the CF and GF to some extent, and then subsequently decreased with a further increase in CF and GF. By mixing with a proper quantity of CFs and GFs, the opposition to climate exhaustion degeneration can be significantly improved. Various applications of GFRP and CFRP composites with the continuous improvement in growth of the market were also discussed. Finally, for improving the properties of the new-developed GFRP and CFRP composites, the fibers should be treated with different chemicals and the matrix blended with proper chemicals for obtaining the GFRP and CFRP composites. Thus, the chemical treatment utilized for both the fibers and the matrix will enhance the mechanical, thermal and tribological characteristics of the GFRP and CFRP composites.

Current progress, new advances and future research directions on FRP composite manufacturing are summarized and presented. However, the continuous demand for composite structures leads to a huge consumption of materials that affect the environment. Certain fibers (e.g., carbon fibers), used to improve properties in various industries, represent a major impediment to recycling at the end of the composites’ life. Therefore, the current environmental situation, which has reached a critical point, requires prompt and objective actions to reduce greenhouse gas emissions. Thus, the orientation of obtaining advanced composites from renewable energy resources would be an optimal ecological solution. Moreover, future research directions may be geared towards recycling existing composites into high-value alternative products. Furthermore, it is necessary to develop new advanced technologies for post-consumer waste management or at least to improve current FRP composite production technologies.

## Figures and Tables

**Table 1:** Chemical composition, characteristics and main applications of glass fibers.

Fiber Category	Composition	Characteristics	Applications
A-Glass	alkali-lime glass with little or no boron oxide	- higher durability and electrical resistivity - not very resistant to alkali	- when alkali resistance is not a requirement - process equipment
C-Glass	alkali-lime glass with high boron oxide content	resistant to chemical attack and most acids which dissolve e-glass	when higher chemical resistance to acid-induced corrosion is required: glass staple fibers and insulation
D-Glass	borosilicate glass	low dielectric constant	when low dielectric constant is preferred
E-Glass	alumino-borosilicate glass with less than 1 wt.% alkali oxides	- not chloride-ion resistant - high electrical resistivity - good strength/stiffness properties - good heat resistance - the lowest cost	- mainly for GFRP composites from transport, building and aeronautics. - originally for electrical (protection of cables, sheaths and pipes) and thermal (sealing for piping, oven doors) applications
AR-Glass		resistant to alkali environment	- when alkali-resistance is required - cement substrates and concrete
R-Glass	alumino-silicate glass without MgO and CaO content	- good mechanical properties - acid corrosion resistance - higher strength	- automotive industry - docks and marinas - applications with high mechanical requirements
S-Glass	alumino-silicate glass without CaO but with high MgO content	- highest tensile - strength among all types of fibers - higher heat resistance - high modulus	- aerospace industry - military aircraft components - missile casings - when high tensile strength required

**Table 2:** Main physical, mechanical, electrical, thermal and optical properties of glass fibers [35,36,37,38,39].

Properties		Type of Glass Fiber							
		A	C	D	E	AR	R	S	S-2
Physical	Density (g/cm^3^)	2.44	2.52–2.56	2.11–2.14	2.54–2.60	2.70	2.54	2.48–2.49	2.46
Mechanical	Tensile Strength (MPa)	3310	3310	2415	3450	3241	4135	4585	4890
	Elongation at Break (%)	4.8	4.8	4.6	4.8	4.4	4.8	5.4	5.7
	Young’s Modulus (GPa)	68.9	68.9	51.7	72.4	73.1	85.5	85.5–46.9	46.9
	Poisson’s Ratio (-)	0.183	0.276		0.200			0.22	0.230
	Shear Modulus (GPa)	29.1	27.0		30.0			35.0	35.0
Electrical	Electrical Resistivity (Ω-cm)	10^8^	-		4.02 × 10^12^		2.03 ×10^12^	9.05 × 10^10^	9.05 × 10^10^
	Dielectric Constant (-)	6.2	6.9	3.8	5.9–6.4	8.1	6.4	5.1–5.34	5.3
	Dissipation Factor (-)	-	0.0085		0.0025			0.0034	
	Dielectric Strength (kV/mm)				10.3			13.0	
Thermal	CTE, linear (μm/m-°C)	9.0	6.3	2.5	5.0	6.5	3.3	5.2–5.6	1.6
	Specific Heat Capacity (J/g-°C)	0.796	0.787	0.733	0.810		0.732	0.737	0.737
	Softening Point (°C)	727.0	750	771	840.6	773	952		1056
	Thermal Conductivity (W/m-K)	-	1.1		1.3				1.45
	Thermal expansion coeff. (×10^−7^)	73	63	25	54	65	33		16
	Melting Point (°C)				≥1725			≥1725	
	Annealing point (°C)		588	521	657				816
	Strain point (°C)		522	477	615		736		766
Optical	Refractive Index (-)	1.538	1.533	1.465	1.558	1.562	1.546	1.525	1.521

**Table 3:** Physical/mechanical properties and main applications of carbon fibers according to precursor material [45].

Fiber Type	Precursor Material	Density (g/cm^3^)	Tenacity (GPa)	Modulus (GPa)	Breaking Extension (%)	Characteristics	Applications
HS	PAN	1.7–1.8	2.8–4	230–250	1.0–2.0	- excellent strength - wear resistance - high dimensional stability - specific toughness - fatigue resistance	- aircraft/aerospace equipment - sporting goods: tennis rackets, softball bats, hockey sticks and archery arrows and bows - automobiles/vehicles - industrial equipment parts
UHS	PAN	1.7–1.8	4.1–5.7	260–290	0.8–1.0	- low thermal expansion - lightweight - ultra-high strength - high stiffness	- aerospace industry: space, military and commercial (military vessels, energy and gas storage) - industrial use - wind turbine blades
LM	Pitch	1.3–1.7	0.6–1.0	40–60	2.0–5.0	- low modulus - lightweight - high stiffness - corrosion resistance - high electrical conductivity - thermal conductivity	- used in stiff and thermally conductive elements - construction and civil engineering: bridges and bridge columns, decks - high-end sporting goods - industry: energy storage (flywheels, pressure vessels), filtration media, thermal management (radiators) - satellite (antenna/refractor)
HM	PAN/Mesophase pitch	1.8–2.0	1.7–3.5	450–750	0.5	- high modulus - lightweight - high stiffness	- attractive for applications where high stiffness and lightweight are required - aerospace industry - sports, robotic arm, machine toll - automotive, transportation, marine
UHM	Mesophase pitch	2.0–2.2	2.1–2.4	600–900	0.2	- ultra-high modulus - lightweight - high stiffness - excellent vibration damping property	- radar and telecommunication applications - sport: competitive golf clubs - industrial equipment: composite rollers, beams for transfer machines - offshore oil exploration and production

**Table 4:** Main mechanical properties of GFRP composites according to different types of reinforcements and matrix.

Reference	Type of GF	Resin	Curing Agent	Volume of Fiber (%)	Process Type	Sample Thickness (mm)	Testing Standard	Tensile Strength (MPa)	Flexural Strength (MPa)	Elongation at Break (%)
[98]	E-glass fiber	Heat-cured acrylic resin	-	-	Pultrusion	2.17 ± 0.15	-	-	265.4	1.000
[99]	E-glass fiber	Bispenol a type epoxy resin	Blend of TEPA and NP)	-	Autoclave	2.5	-	270	-	-
[100]	Randomly Oriented	Epoxy (10 wt.% Sic)	-	4.817	Hand lay-up	-	ASTM D 3039-76 (T), D 256 (I)	179.4	297.82	-
[101]	Woven mat	Polyester	-	-	-	-	ASTM D 638-97 (T)	249	-	-
[102]	E-glass fiber	epoxy resin (lycal type)	-	61	Hand lay-up	2.96	ASTM D3039/D3039M-17	260.98	-	6
[103]	Unidirectional	Epoxy	-	55	Hand lay-up	2	ASTM D3039 (T)	784.98		0.032
[104]	Woven + (35 wt.% Short Borosilicate)	Epoxy	-	-	Hand lay-up	1	-	355		1.65
[105]	Chopped strand Mat	Polyester	-	60	Hand lay-up	0.1 ± 0.005	ASTM D638 (T)	250	-	0.022
[106]	Woven glass fabrics	Epoxy resin	Polyamine	-	Dry Hand lay-up	3	ASTM D 638	205.1	-	3.30
[107]	E-glass fiber	Epoxy	Hardener	6.88	Molding process	3	ASTM G76	516	393.1	-
[108]	Plain-woven fabric	Epoxy resin	Amino based hardener	-	Vacuum bagging	3	ASTM 3039-08, D790-10, D256-10, D3039-08	278.38	319.50	-
[109]	Chopped Strand + verticalRoving	Polyester	-	-	-	-	ASTM D 3039, D 5379	103.472	-	-
[110]	Chopped strand	Epoxy	Hardener	4.2	Hand lay-up	75	ASTM C618-99, D695-96	-	-	-
